# Transcriptional landscape of the pMP7017 megaplasmid and its impact on the *Bifidobacterium breve* UCC2003 transcriptome

**DOI:** 10.1111/1751-7915.14405

**Published:** 2024-01-11

**Authors:** Rebecca L. Dineen, Francesca Bottacini, Mary O'Connell‐Motherway, Douwe van Sinderen

**Affiliations:** ^1^ APC Microbiome Ireland University College Cork Cork Ireland; ^2^ School of Microbiology University College Cork Cork Ireland; ^3^ Department of Biological Sciences Munster Technological University Cork Ireland

## Abstract

The 190 kb megaplasmid pMP7017 of *Bifidobacterium breve* JCM7017 represents the first conjugative and largest plasmid characterised within this genus to date. In the current study, we adopted an integrated approach combining transcriptomics, whole genome comparative analysis and metagenomic data mining to understand the biology of pMP7017 and related megaplasmids, and to assess the impact of plasmid‐carriage on the host strain. The data generated revealed variations within basic features of promoter elements which correlate with a high level of transcription on the plasmid and highlight the transcriptional activity of genes encoding both offensive and defensive adaptations, including a Type IIL restriction‐modification system, an anti‐restriction system and four Type II toxin‐antitoxin systems. Furthermore, a highly transcribed tmRNA, which likely provides translational support to the host strain, was identified, making pMP7017 the first plasmid of the *Bifidobacterium* genus and the smallest plasmid known to express a tmRNA. Analyses of synteny and variability among pMP7017 and related plasmids indicate substantial diversity in gene organisation and accessory gene cargo highlighting diverse (co‐)evolution and potential host‐specific rearrangements and adaptations. Systematic analysis of the codon usage profile of transcriptionally active pMP7017‐encoded genes suggests that pMP7017 originated from (sub)species of *Bifidobacterium longum*. Furthermore, mining of metagenomic data suggests the presence of pMP7017‐homologues in ~10% of microbiome samples, mostly infants and/or mothers from various geographical locations. Comparative transcriptome analysis of the *B. breve* UCC2003 chromosome in the presence or absence of pMP7017 revealed differential expression of genes representing 8% of the total gene pool. Genes involved in genetic information processing were exclusively upregulated, while altered expression of genes involved in biofilm production and polysaccharide biosynthesis was also observed.

## Graphical abstract

An in‐depth analysis of bifidobacterial megaplasmid biology revealed novel features, evidence of diverse (co‐)evolution and potential host‐specific rearrangements and adaptations among pMP7017‐related megaplasmids. The presence of pMP7017‐homologues in ~10% of human faecal samples from various geographical locations reinforces the relatively high prevalence of this megaplasmid family. Alteration of the hosts transcriptional profile upon pMP7017 acquisition demonstrates complex megaplasmid‐host interactions.

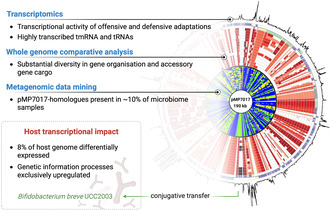

## INTRODUCTION

*Bifidobacterium* is a genus of high GC% gram‐positive (G+) anaerobic bacteria, characterised by their Y‐shaped or ‘bifid’ morphology from which they derive their name (Tissier, 1990). Bifidobacteria are ubiquitous inhabitants of the gastrointestinal tract of mammals and constitute one of the dominant genera of the intestinal microbiota. They possess diverse saccharolytic capabilities with an estimated 13.7% of their protein‐coding capacity dedicated to carbohydrate metabolism, which is presumed to be essential for their lifestyle and species‐specific niche adaptions (Milani et al., 2016). Probiotic strains belonging to *Bifidobacterium* spp. have been exploited in the food industry, as prophylactics and therapeutic agents, due to their contribution to the maintenance of a balanced microbiota and the health‐promoting attributes with which they have been associated (Abdolalipour et al., 2020; Alessandri et al., 2019; Arboleya et al., 2016; Esteban‐Torres et al., 2021; Faghfoori et al., 2021; Fanning, Hall, Cronin, et al., 2012).

While plasmids are common among many intestinal bacterial species, plasmids of bifidobacterial origin were considered rare, with only small cryptic plasmids identified in five species (Álvarez‐Martín et al., 2007; Iwata & Morishita, 1989; Matteuzzi et al., 1990; Moon et al., 2009; O'Riordan & Fitzgerald, 1999; Park et al., 1997, 2008; Sgorbati et al., 1982, 1986). However, the discovery of a 190 kb conjugative megaplasmid, pMP7017, in host strain *Bifidobacterium breve* JCM7017 and the subsequent identification of pMP7017‐homologues in 15 *Bifidobacterium longum* subsp. *longum* strains highlighted the presence of large plasmids within this genus (Bottacini et al., 2015; Odamaki et al., 2018; Schwartz, 2009).

Horizontal gene transfer meditated by conjugative plasmids is considered an important driver of genome variability and ecological adaption, due to the transfer of accessory genes between different host strains, while also providing a reservoir of genetic variation offering bacteria an excellent platform for rapid functional and evolutionary innovations. More recently, plasmids have been linked to the altered expression of host genes and the manipulation of bacterial phenotypes, with increasing evidence suggesting that plasmid‐mediated alterations to host gene expression may affect niche‐adaptive fitness (Coulson et al., 2015; Ghigo, 2001; Lang & Johnson, 2015; Matsumoto et al., 1998; Parashar et al., 2013; Ranjan et al., 2018; Vial & Hommais, 2020; Weber et al., 2015).

As such, ‘omics’ approaches are fundamental in disentangling the plasmid‐host relationship and shedding light on the potential regulatory networks, costs of plasmid carriage and potential fitness trade‐offs. Furthermore, analysis of transcriptomics data, constituting the complete set of RNAs transcribed from a genome at a particular time‐point in a given environment, generates indispensable information on gene functionality, level of expression and regulation.

While transcription in bacteria can be modulated in a number of ways, via *cis*‐acting elements which alter the affinity of the RNA‐polymerase (RNAP) holoenzyme for the promoter or by *trans*‐acting regulatory elements which may prevent or enhance promoter binding, variability within the basal features of promoters underpins all transcription regulation (Browning & Busby, 2004). Bacterial promoters are modular in structure, consisting of two RNAP recognition sites, these hexameric sequences are located at the −10 and −35 positions upstream of the first transcribed base (+1), known as the transcriptional start site (TSS), and are separated by a spacer of 15–19 bps whose sequence is generally not conserved. As observed for other bacteria, promoter strength in bifidobacteria has been indicated to correlate with the degree of identity to the consensus −10 and −35 sequences, as well as the length of the spacer (Bottacini et al., 2017). Comparative analysis of promoter regions allows the identification of sequence variations associated with promoter activity and how these variations impact on the corresponding transcription levels.

Further to promoter strength and regulatory mechanisms, the genome‐wide effects of codon usage on transcription levels have been demonstrated, essentially this code within a code may not only affect peptide elongation rates but also influence transcription levels by modulating transcription efficiency and mRNA stability (Zhou et al., 2016). As the coevolution of intracellular tRNA abundance and codon usage is essential in the optimisation and efficient use of cell resources, tRNAs may therefore constitute dynamic rather than static contributors to gene expression and the codon landscape.

In the current study, we used an integrated approach of transcriptomic data analysis and functional characterisation of plasmid pMP7017 to interpret its functional genetic elements and their associated transcription levels. To elucidate the biological function and importance of these expressed genes we utilised a variety of sequence and structural homology‐based tools. Further to this, we investigated if the codon usage profile of transcriptionally active genes correlates with their expected expression levels and how this relates to overall tRNA abundance. In addition, transcriptional data sets pertaining to the *B. breve* UCC2003 chromosome in the presence or absence of pMP7017 were compared to uncover the transcriptomic impact of pMP7017 acquisition by the host cell. Finally, we explore the prevalence, synteny and variability of pMP7017 and related megaplasmids, through comparative analysis of whole genome sequences and mining of metagenomic data.

## EXPERIMENTAL PROCEDURES

### RNA isolation

*Bifidobacterium breve* UCC2003 cultures grown in MRS (Difco) supplemented with cysteine HCl and tetracycline until an OD_600nm_ of 0.6 was reached, the cells were then centrifuged at 10,000×*g* for 2 min at RT. The obtained cell pellet was suspended in 1 mL of QIAZOL (Qiagen, United Kingdom) and placed in a tube containing 0.8 g of glass beads (diameter, 106 μm; Sigma). The cells were lysed by shaking the mix on a BioSpec homogeniser at 4°C for 2 min (maximum setting). The mixture was then centrifuged at 12,000 rpm for 15 min, and the RNA‐containing upper phase was recovered. RNA was further purified by phenol extraction and ethanol precipitation. The quality and integrity of the RNA was checked by the Tape station 2200 (Agilent Technologies, USA) analysis. RNA concentration and purity were evaluated by Picodrop microlitre Spectrophotometer (Picodrop, UK). Three independent biological replicates for each condition described in this work were performed.

### RNA sequencing

2.5 μg of total RNA was treated by the Ribo‐Zero Magnetic kit (Illumina) to remove ribosomal RNA, followed by purification of the rRNA‐depleted sample by ethanol precipitation. RNA was further processed according to the manufacturer's instructions. The yield of rRNA depletion was checked by Tape station 2200 (Agilent Technologies). Then, 400 ng of the rRNA‐depleted RNA sample was fragmented using a Bioruptor NGS ultrasonicator (Diagenode, USA) followed by size evaluation using Tape station 2200 (Agilent Technologies). A whole transcriptome library was constructed using the TruSeq Stranded RNA LT Kit (Illumina). Samples were loaded into a Flow Cell V3 150 cycles (Illumina) according to the manufacturer's instructions. The reads were depleted of adapters, quality filtered (with overall quality, quality window and length filters) and aligned to the *Bifidobacterium* and plasmid reference genomes through the Burrows‐Wheeler Aligner (Li & Durbin, 2009). Counting of reads whose sequences correspond to open reading frames (ORFs) was performed using HTSeq (http://htseq.readthedocs.io/en/release_0.9.1/) and analysis of the reads per kilobase per million was mapped. RPKM values were performed using the following formula (numReads = number of reads [fragments] mapped to a gene sequence, geneLength = length of the gene sequence and totalNumReads = total number of mapped reads of a sample):

$$\text{RPKM}=\frac{\text{numReads}\;}{\frac{\text{geneLength}}{10^{3}}\times \frac{\text{totalNumReads}}{10^{6}}}.$$

While the accepted threshold values for expression range in the literature from ≥1 up to ≥2 RPKM, a higher value was chosen in this work to minimise false positives, assigning less weight to false negatives for this analysis. Consequently, a RPKM threshold value for expression of ≥1.5 RPKM was established, as determined by a density plot of the normalised RPKM values. We then calculated the quantile values (Q1, Q2 and Q3) of the normalised gene expression data to establish thresholds for levels of transcription, low, medium or high (Figure S9).

### Identification of promoters and transcriptional terminators

For each expressed gene and operon transcript boundaries were identified from the RNAseq data, following quality trimming (threshold >20, fraction of low‐quality bases <0.1) and subsequent mapping, as the first and last base of reads where an increase or drop in sequence coverage was observed, respectively. A region of 62 bps upstream of each identified TSSs was first searched using Meme Suite (http://meme‐suite.org/) to identify the best fit to a canonical −10 and −35 promoter sequence (Bailey & Elkan, 1994). A promoter list was then generated from the obtained predicted canonical promoter and re‐aligned with Meme to ensure the correct positioning of the −10 and −35 hexamers, before being manually annotated in Artemis (http://www.sanger.ac.uk/science/tools/artemis). The WebLogo web server (https://weblogo.berkeley.edu/) was then used to visualise the level of conservation between 25 and 30 aligned promoters of highly and moderately transcribed TUs, respectively (Crooks et al., 2004).

Rho‐independent transcriptional terminators were first predicted using ARNold and manually refined in Artemis (http://www.sanger.ac.uk/science/tools/artemis) (Rutherford et al., 2000). Meme Suite (http://meme‐suite.org/) was used to identify stem‐loop structures and the strength of each GC‐rich stem‐loop was confirmed using the RNAfold webserver (http://rna.tbi.univie.ac.at/cgi‐bin/RNAWebSuite/RNAfold.cgi) (Gruber et al., 2008). Where appropriate, terminators in addition to the ones predicted above were included following a manual search for the presence of polyT stretches downstream of putative stem‐loop structures at the end of transcripts.

As there is a known discrepancy between true TSSs and those identified by RNAseq, where RNAseq TSSs tend to be missing bases (~20 bp) at the 5′‐ untranslated region (UTR), following promoter and terminator identification, transcript boundaries were manually refined in order to more accurately establish 5′ and 3’ UTR length (Bottacini et al., 2017; Wang et al., 2015).

### Prediction of RNAs

Prediction of tRNAs, sRNAs and RNA‐based regulatory elements was performed using the RNAspace web server (http://www.rnaspace.org/) and the structRNAfinder webserver (https://structrnafinder.integrativebioinformatics.me/) (Arias‐Carrasco et al., 2018; Cros et al., 2011). Sequences producing hits with both webtools were then cross‐referenced and verified using the Rfam database (http://rfam.xfam.org/) (Griffiths‐Jones et al., 2003). Identified tRNAs which had conflicting annotated amino acid specificities were annotated as ‘tRNA_OTHER’.

### Indices of codon usage

Multivariate analysis of codon usage and the corresponding indices of codon usage for all datasets were determined using the program CodonW (J Peden, version 1.4.2 http://codonw.sourceforge.net/). The relative synonymous codon usage (RSCU) is calculated as the observed frequency of a codon divided by the expected frequency. RSCU values range from 0 (codon absent), through 1 (no bias) to 4 (single codon used out of a four‐codon family), that is values >1 indicate that a codon was used more frequently than expected, where the converse is true for values <1. The Codon Adaptation Index (CAI) and Codon Bias Index (CBI) were used to estimate the extent of bias towards codons preferred in highly expressed genes and those of the host strain genome. CAI values range from 0 to 1.0, an increase in CAI values positively correlates with an increase in codon usage bias and thus expression level or the host strain.

### Generation of transconjugants

Individual overnight cultures of donor and recipient strains were inoculated at 1% (v/v) into mMRS broth supplemented with cysteine‐HCl (0.05% [wt/vol]) and lactose (1.0% [wt/vol]), and grown to an optical density (OD600 nm) value 0.8–1.0 at 37°C under anaerobic conditions. Donor and recipient cells were combined in a 1:1 ratio, based on OD value, up to a total volume of 2 mL, briefly vortexed and concentrated by centrifugation at 2200×*g* for 5 min. The resultant cell pellet was resuspended in 200 μL of RCM and spread‐plated onto non‐selective agar. RCA plates were incubated at 37°C overnight under anaerobic conditions. Following incubation, the donor/recipient conjugation mixture was recovered from the plates using a cell scraper (Sarstedt, Germany) and resuspended in 2 mL of phosphate‐buffered saline and serially diluted. Transconjugants were isolated based on the selective carbohydrate for recipient cells in combination with tetracycline (10 μg mL^−1^) to select for the presence of a pMP7017‐derivative that had been marked with a tetracycline resistance gene. For validation purposes, presumed transconjugants were grown in mMRS broth with tetracycline at a concentration of 10 mg mL^−1^ and the appropriate carbohydrate, after which DNA was isolated and PCR performed with primers based on genes unique to the donor strain, recipient strain and the megaplasmid.

### Differential expression of *B. breve* UCC2003 chromosomal genes in the presence of pMP7017

Two‐way hierarchical clustering was performed using transcriptional data of *B. breve* UCC2003 chromosomal genes in the presence and absence of pMP7017 based on transcripts per million (TPM), using the following formula:

$$\mathrm{TPM}=10^{6}\times \left(\frac{\left(\frac{\text{reads mapped to gene}}{\text{gene length}}\right)}{\mathrm{Sum}\left(\frac{\;\text{reads mapped to gene}}{\text{gene length}}\right)}\right).$$

The gene expression dataset generated derives from three independent biological replicates of each pMP7017‐containing and pMP7017‐free *B. breve* UCC2003 cells. From this data, a heatmap with K‐means clustering was generated. Using the sum of squared error (SSE) the optimum number of clusters was determined to be two, containing a total of 157 differentially expressed genes (DEGs), represented by 30 down‐regulated and 127 upregulated genes, after filtering based on mean expression, fold change (fc) ≥2 and a *p*‐value of <0.05 with a false discovery rate (FDR) cut‐off of *p* < 0.1 for each compared pair.

Protein families and pathway assignments were achieved utilising the BioCyc (https://biocyc.org/) and kyoto encyclopedia of genes and genomes (KEGG) databases (https://www.genome.jp/kegg/) while enrichment analysis was performed using ShinyGO (Ge et al., 2020; Kanehisa et al., 2002; Karp et al., 2019). Enrichment analysis is a method of functional class scoring which utilises the log2 fcs for all genes to determine whether gene sets for particular biological pathways are enriched among large positive or negative fcs in transcription within the differentially expressed gene dataset relative to the reference genome.

### Comparative analysis

Putative pMP7017 protein sequences were aligned using Diamond with an *e*‐value cut‐off of 0.0001, >50% sequence coverage and >50% of identity cut‐off (Buchfink et al., 2015). The R package, circlize, was then employed to produce a circular heatmap (Gu et al., 2014), illustrating the percentage of identity of each pMP7017 proteome against 43 replicase‐positive Refseq strains (Tatusova et al., 2016). Search of pMP7017 genes across reads obtained from 1846 publicly available infant metagenomes (Table S11) datasets was performed using Bowtie2 and normalised using RPKM (Langmead & Salzberg, 2012). Reassembly of metagenomes containing megaplasmid‐associated genes was conducted using metaSPAdes (Nurk et al., 2017).

## RESULTS

### Transcription‐mapping reveals features of DNA regulatory elements which correlate with transcription levels in pMP7017

To identify genes and operons that are actively transcribed in pMP7017 during logarithmic growth and to connect features of promoter elements with levels of transcription, we analysed the pMP7017 transcriptome and assessed the basic features of transcriptionally active promoters and transcription termination sites, employing a combination of high‐throughput RNA sequencing (RNAseq) analysis and promoter mapping.

**FIGURE 1:**
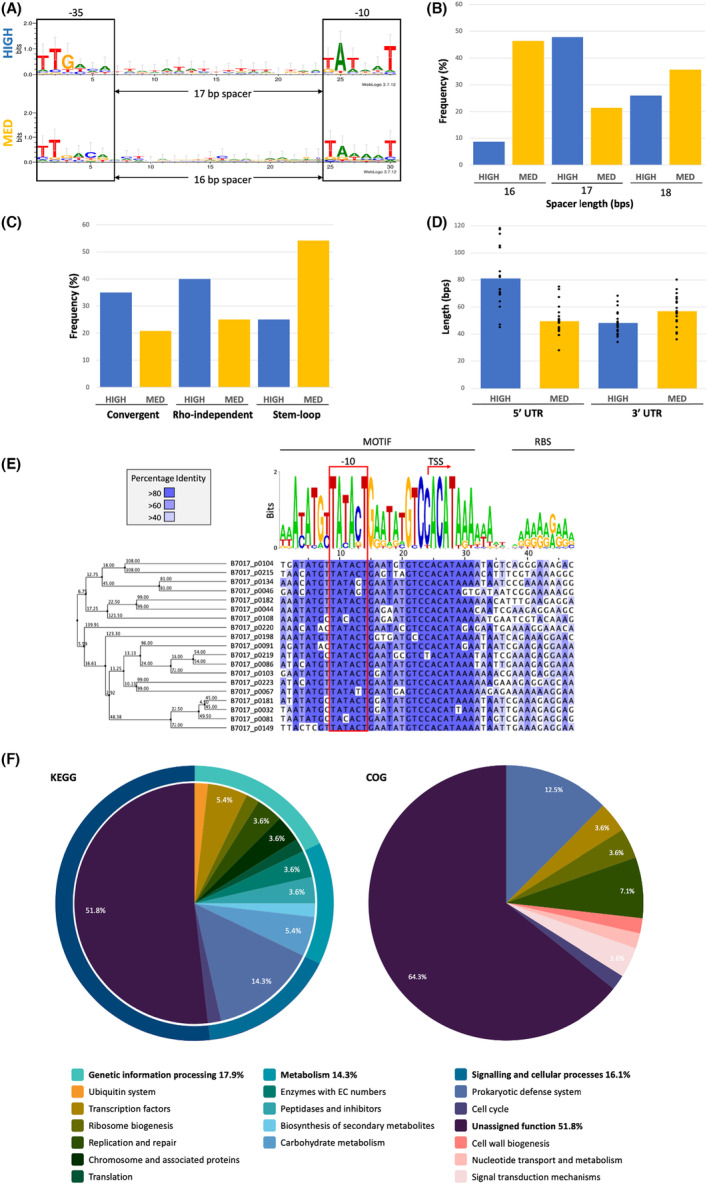
Intrinsic features of pMP7017 promoters, transcriptional unit elements and termination signals, as a function of transcriptional rate. (A) Sequence logo showing the consensus motifs identified for the −10 and −35 sequences of the canonical promoter in pMP7017 across the levels of expression high and medium‐low. (B) Bar chart representing the optimal spacer length of highly expressed and medium expressed genes. A spacer of 17 bp is more prevalent in highly expressed genes, while a spacer of 16 bps has the highest occurrence in the promoters of genes with a lower level of expression and 17 bps is less frequently observed. (C) Bar plot showing the different strategies for transcriptional termination identified in pMP7017, grouped by transcription level (high vs. medium). The bar chart suggests a higher prevalence of rho‐independent termination in highly transcribed genes. (D) Bar plot comparing the length of the 5’‐UTR and 3’‐UTR regions of genes grouped by transcription level high versus medium. From the chart, it is possible to observe a higher average 5’‐UTR length in highly transcribed genes as compared to gene with a lower level of transcription. (E) Sequence logo (top) highlighting the conserved residues among the 19 identified motifs, −10 promoter element (boxed in red), presumed transcriptional start sites and downstream ribosome binding sites. The corresponding sequence alignment (below) is coloured based on the percentage of nucleotide identity (40%–100%). The phylogenetic relationship of the given sequences is depicted in the unrooted phylogram based on the average distances of the DNA with bootstrap values (left). (F) Pie chart representing the relative abundances of COG and KEGG functional categories within the presumed pMP7017‐specific regulon. COG, clusters of orthologous groups; KEGG, kyoto encyclopedia of genes and genomes.

Based on our results, highly transcribed genes of pMP7017 possess a promoter that best resembles the accepted canonical bifidobacterial consensus (TTGACAn_17_TATAAT) (Bottacini et al., 2017). The highest level of conservation observed within these motifs are residues A in the 2nd position and T in the 6th position of the −10 box, T in the 2nd position and G in the 3rd position of the −35 box, with an optimum spacer of 17 bps (Figure 1A). When comparing promoter sequences from highly transcribed versus those from moderately transcribed TUs we observed a more degenerate promoter consensus in the latter case, in addition to a higher occurrence of a ±1 deviation from the optimal spacer distance of 17 bps (Figure 1B). This data is consistent with similar transcriptional profiling and promoter analyses of the *B. breve* UCC2003 genome (Bottacini et al., 2017).

Analysis of the approximate 3′ end of transcripts based on the obtained RNAseq data suggests that rho‐independent termination represents the more frequently adopted method of transcription termination of highly transcribed genes and operons by pMP7017 (Figure 1C). Convergent transcriptional termination is the second most frequent, whereby RNA polymerase conflicts result in efficient bidirectional termination which marks transcript boundaries, a method of transcription termination which is considered pervasive in bacteria (Ju et al., 2019). Transcriptional units with moderate levels of expression appear to favour stem‐loop structures, mRNA hairpins characterised as GC‐rich inverted repeat sequences. These structures differ from rho‐independent termination signals due to the absence of a poly U tract (in nascent RNA) and have been suggested as intrinsic termination signals as they can allosterically alter the structure of the RNA polymerase resulting in its release (Gupta & Pal, 2021). In addition, examination of the 5′ and 3′ UTRsof highly expressed versus moderately expressed genes indicate that the 5’‐UTR of highly expressed genes tends to be longer on average compared to those of moderately expressed genes which may serve to stabilise the transcript or indeed constitute regulatory functions (Figure 1D). We then analysed the genomic regions encompassing the promoters and 5’‐UTRs of highly expressed genes to assess for potential regulatory motifs, revealing a conserved 34 bp motif overlapping the −10 promoter element of six genes (5′‐AW**A**Y**ATG**Y **TA**T**A**C**T** **G**AATA**TG**T**C**C**ACAT**AAAAWA‐3′, 100% conserved residues in bold, −10 underlined). We then used the consensus motif as bait across a 60 bp region upstream of all presumed translation start sites, revealing 13 additional motifs distributed throughout the plasmid (Figure 1E). Based on the transcriptional data and promoter mapping in this study, these promoters are predicted to drive the expression of 56 genes, representing ~24.1% of pMP7017's coding capacity. In an effort to establish commonality of function within this presumed regulon, all genes were grouped into functional categories based on the clusters of orthologous groups (COG) and KEGG classification systems (Figure 1F; Table S1). While the role(s) of 51.8% of these genes remains to be determined, the remaining 48.2% representing 27 genes fall within several functional categories, predominantly and ordered by relative abundance: prokaryotic defence systems, replication and repair, transcription and carbohydrate metabolism. As the conserved 34 bp sequence appears to overlap the −10 promoter element, this motif likely represents a transcription factor binding site, the binding of which may inhibit or enhance transcription. Sequence‐homology‐based analysis revealed the presence of this motif preceding comparable protein coding sequences within presumed pMP7017‐homologues, yet this motif is absent among bifidobacterial chromosomes suggesting that it is unique to pMP7017 and related plasmids.

### Transcriptional activity of pMP7017‐genes encoding offensive and defensive systems likely ensure its maintenance and enhance the success of its lateral transfer

While over half of the predicted gene content of plasmid pMP7017 is considered transcriptionally active at the mid‐logarithmic growth phase of *B. breve* UCC2003, 54.2% of these expressed genes are currently functionally unassigned (Figure S1).

Comprehensive computational analysis of each pMP7017 coding sequence enabled us to assign a putative functional role to an additional 15 genes and further refine the functional annotation of 21 transcriptionally active genes (Table S2). In addition, we identified four previously unassigned, transcriptionally active Type II Toxin‐Antitoxin systems, which fall within three toxin superfamilies, AtaT/TacT, HipAB and two systems of the ParE/RelE superfamily, all of which are predicted to act through translation elongation inhibition in plasmid‐free cells promoting post‐segregational killing (Table S3).

The highest level of gene transcription was observed for transposable elements, genes specifying replication functions, Type‐II TA systems and genes encoding predicted surface‐associated proteins. Genes predicted to specify multimer resolution functions, additional recombinases and active partitioning systems were expressed at moderate levels, ensuring the structural and segregationally stability of the plasmid. Moderate levels of transcription were also observed for genes encoding putative defence systems, including a homologue of the so‐called Lar anti‐restriction protein and a homologue of the Type‐IIL restriction modification (RM) enzyme MmeI, corresponding to locus tags B7017_p0162 and B7017_p0174, respectively (King & Murray, 1995; Morgan et al., 2008). While a low level of transcription was observed for locus B7017_p0197, encoding a presumed N6_N4_Mtase.

pMP7017 was also shown to contain Type IE clustered regularly interspaced short palindromic repeats (CRISPR) and encode their associated (*cas*) genes which together constitute an RNA‐based adaptive immunity system (Bottacini et al., 2015). However, transcriptional activity above the assigned threshold was observed solely for the CasE‐specifying gene, a protein involved in spacer acquisition when complexed with Cas1 and an internally located hypothetical gene (locus tag: B7017_p0086). Furthermore, transcription of the associated CRISPR arrays (above our assigned threshold) was not detected, suggesting the pMP7017 CRISPR/*cas* system is non‐functional or dormant. Nevertheless, CRISPR spacers that are inserted between direct repeats in CRISPR arrays represent a biological record of past encounters with foreign DNA, as such CRISPR spacers provide a valuable source of information regarding invading elements‐host interactions. Characterisation of the pMP7017 spacer sequences revealed two complete matches of spacers 10 and 18 to *Siphoviridae* bacteriophage genomes and partial matches (>45% identity) for 17 of the 25 remaining spacers to various actinobacterial phage sequences (Table S4).

### Identification of pMP7017‐encoded RNAs

In silico characterisation of pMP7017 upon its discovery indicated the presence of 14 putative transfer RNA (tRNA) genes flanking the pMP7017 replicase‐encoding gene (Bottacini et al., 2015; Dineen et al., 2021). Analysis of the transcriptional data of pMP7017 verified the presence of a tRNA array unit at the previously indicated genomic locus, however upon careful inspection this region was found to contain a total of 20 tRNA‐specifying genes organised within three tandemly arranged TUs. The data further highlights the high level of transcription for 17 of the 20 tRNA genes, which collectively correspond to 12 amino acids. To verify that the observed level of transcription was not due to mismapped chromosomal tRNA transcripts, pMP7017 tRNA gene sequences were aligned with the corresponding host‐encoded tRNA genes revealing significant divergence, thus ensuring the observed level of transcription was a result of transcripts mapping uniquely to pMP7017‐encoded tRNAs (Table S5).

Furthermore, analysis of the pMP7017 transcriptome allowed the identification of two non‐coding RNAs (ncRNAs) not previously reported. The first of these is a highly transcribed 397 bp transfer messenger RNA (tmRNA), which is located between the replicase‐encoding gene and the tRNA region (nucleotide coordinates 120,876‐121,272). tmRNAs are bifunctional chimeric molecules involved in the rescue of stalled ribosomes during translation, while they also function in targeting truncated polypeptides for degradation, a system known as trans‐translation (Janssen & Hayes, 2012). The pMP7017 tmRNA (tmRNA^pMP7017^) single chain 397 bp mature transcript encodes the tag peptide A^RESUME^NTVSSSRFTLAA‐ which is recognised by DDE/DHE proteases for proteolytic cleavage of polypeptides produced from truncated mRNAs.

In addition, a 178 bp *manA* motif was identified (nucleotide coordinates 122,656‐122,830), this conserved cis‐regulatory RNA structure is thought to function as a riboswitch due to its complex architecture (Weinberg et al., 2009). This motif is most commonly found within the 5’‐UTR of genes involved in mannose metabolism or adjacent to tRNAs in some cases, and while its function is not well defined, *manA* is associated with the regulation of genes involved in mannose metabolism, nucleotide synthesis and stress response (Weinberg et al., 2010).

### pMP7017‐carriage of tRNA genes facilitate configuration of the cellular tRNA pool to suit the codon usage of plasmid‐encoded genes

Empirical patterns of synonymous codon usage, codons that specify the same amino acid, are not used in equal frequencies across species, genomes or individual genes. This variation has been mainly attributed to mutation biases and adaptation of codon usage to cellular tRNA abundance (Plotkin & Kudla, 2011). Highly expressed genes generally present with an optimal codon usage profile corresponding to enriched tRNA species, as genes which are highly transcribed have been subjected to stronger purifying selection (Liu et al., 2021). The translation efficiency of plasmid‐encoded genes is thus predominantly determined by its codon bias and the host tRNA pool which impacts the success of both its maintenance and transfer (Tuller, 2011). Horizontally transferred genes with a codon bias deviating from that of the host may sequester ribosomes on plasmid‐borne gene transcripts due to reduced ribosome decoding rates, and as a consequence, the host strain may incur fitness cost due to decreased ribosome availability (Callens et al., 2021; Tuller, 2011). However, plasmids can mitigate these restrictions via the carriage of tRNA genes which have been speculated to facilitate the adaption of the host tRNA pool to the codon usage of plasmid genes, enabling the plasmid to possess a codon bias which in itself would have been mal‐adapted to certain host backgrounds (Tuller et al., 2011).

Following the identification of 17 highly transcribed pMP7017‐encoded tRNAs, we aimed to establish if the codon usage signature of pMP7017 correlates with the tRNA‐species it expresses and to assess how changes in global tRNA abundance due to pMP7017‐expressed tRNAs may impact the host. Analysis of the codon usage profile of all predicted coding sequences of pMP7017 based on the Relative Synonymous Codon Usage (RSCU) values, we observed that the nucleotide composition at the third codon position favour Cytosine (C) or Guanine (G) in the third synonymous codon position, with a distinct preference towards C at the wobble position for both data sets, consistent with what has been observed for bifidobacterial genomes (Figure S2).

**FIGURE 2:**
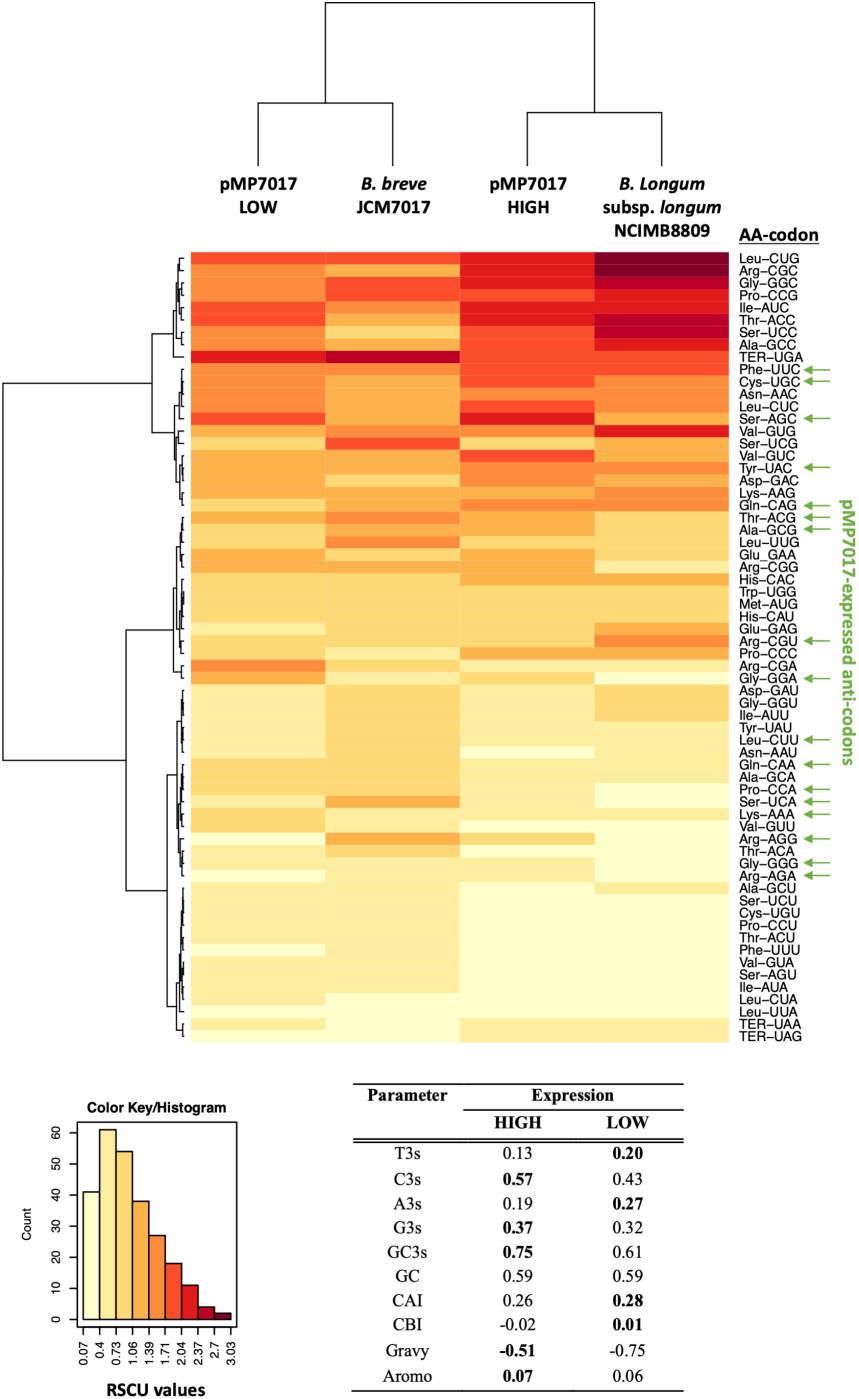
Correlation analysis of transcription level with overall nucleotide composition. A bi‐clustering heatmap of RSCU values from *Bifidobacterium breve* UCC2003, *Bifidobacterium longum* subsp. *longum* NCIMB8809 and pMP7017 (differentiated by level of transcription, LOW vs. HIGH) genomes, using Euclidean distance and average linkage clustering module are shown. High RSCU values, suggesting more frequent codon usage, are represented with darker shades of red, while lower usage frequencies are represented by light colours (as indicated in the colour key). Codon usage parameters of the pMP7017 genome (LOW vs. HIGH expression) are tabulated, where A3, T3, C3 and G3 = frequency of each individual base A, T, C and G, respectively, at the third position of codons. GC3 = Guanine + Cytosine (G + C) content in the third position of codons, GC = total GC content of the entire gene, CAI = Codon Adaptation Index, CBI = Codon Bias Index, Gravy = grand average of hydropathy index, Aromo = aromaticity index.

To ascertain if a correlation could be observed between codon usage patterns and observed levels of transcription of individual pMP7017 genes, the RSCU profile of highly transcribed genes and non‐transcribed genes (those below the assumed FKPM threshold) at mid‐logarithmic growth of host strain *B. breve* UCC2003 were compared. However, two‐dimensional clustering of the normalised data did not distinguish a clear pattern between groups (Figure S3). We subsequently assessed the overall codon usage biases of highly transcribed genes versus those with a low level of transcription to determine if a correlation exists between the level of transcription and overall nucleotide composition. Highly transcribed genes were determined from this analysis to possess an overall GC content at the wobble position (GC_3_) that is 14% higher than those with a low level of transcription (Figure 2). This bias towards G + C pairing in the third codon position of highly transcribed genes may have implications for downstream processes, as a high GC_3_ content has been suggested to impact translation efficiency through enhanced mRNA stability, improved translation fidelity and increased peptide‐elongation rates, ultimately culminating in increased protein production (Gouy & Gautier, 1982). Furthermore, marginally higher values of adaption based on CAI and CBI indices (see Experimental Procedures section) were observed for coding sequences of genes with a lower rate of transcription, while both datasets encode proteins with comparable properties based on *Gravy* and AROMA indices.

Taken together and as expected, these data indicate that highly transcribed genes have been subjected to stronger purifying selection but are less adapted towards the host strain *B. breve* JCM7017 and overall tRNA abundance. Further to this, highly transcribed pMP7017 genes possess a codon usage profile that is similar to that of *B. longum* subsp. *longum* NCIMB8809 (Figure 2). In addition, this data highlights several highly transcribed pMP7017‐encoded tRNAs which correspond to rare or under‐utilised codons in both the host and within the pMP7017 proteome. This may enable the codon usage of pMP7017 to be less dependent on the host tRNA pool, and/or indeed, enhance the translation speed and fidelity of specific mRNAs enriched for these codons.

We subsequently investigated the relationship between pMP7017‐encoded tRNAs, its associated codon usage patterns and the codon usage patterns of the host strain and *B. longum* subsp. *longum* NCIMB8809 genome. This analysis highlights a subset of pMP7017‐expressed tRNAs which specify rare/low‐usage codons (those used at <10% frequency) in the *B. breve* JCM7017 genome, specifically those corresponding to Arg‐AGA, Arg‐AGG, Gly‐GGG, Gly‐GGA, Leu‐CTT, Pro‐CCA and Ser‐TCA (Table S6). Upon comparing the codon usage signatures based on the RSCU values, of the host strain *B. breve* JCM7017 and pMP7017, it is evident that the codon usage of pMP7017 appears to be optimised towards the pMP7017‐encoded tRNA pool (*p*‐value based on *χ* ^2^ test: 0.0013) (Figure S4A). Furthermore, when comparing the codon usage signatures based on the RSCU values of highly transcribed genes to the overall codon usage pattern of pMP7017, highly transcribed genes contain a higher frequency of pMP7017‐encoded tRNA anti‐codons than that observed for the overall pMP7017 gene pool, although this is not of statistical significance (Figure S4B).

These findings suggest that pMP7017‐carriage of tRNA genes facilitates the adaption of the host tRNA pool to the codon usage of pMP7017 genes, while pMP7017‐expressed tRNAs that specify for rare/low‐usage codons may alleviate ineffective translation and enhance expression of target genes in the host.

### pMP7017‐mediated differential expression of *B. breve* UCC2003 chromosomal genes

While the molecular mechanisms underlying the cost and compensation of plasmid carriage are not well understood, recent analyses of transcriptomics data, experimental evolution and mutational analyses have shed light on the specific genetic interactions between plasmids and associated host chromosomes (Ares‐Arroyo et al., 2022; Hall et al., 2021). To evaluate how pMP7017‐carriage may influence the host based on transcriptional differences, transcriptional data of *B. breve* UCC2003 chromosomal genes in the presence and absence of pMP7017 were compared.

From this data, a heatmap with K‐means clustering was generated indicating 157 differentially expressed genes in the presence of pMP7017, represented by 30 down‐regulated and 127 upregulated genes (Figure S5). Functional hierarchical classification based on KEGG Orthology (KO) groups within *B. breve* UCC2003 specific pathways indicates that down‐regulated genes fall within eight protein families, predominantly polysaccharide biosynthesis, membrane transporters and extracellular structures, in addition to the metabolism of amino acids, fatty acids and carbohydrates, as well as genes representing hypothetical functions and mobile elements (Figure S5; Table S7). Gene enrichment analyses of these down‐regulated genes, using the *B. breve* UCC2003 total gene pool as a background, suggest an enrichment of glycosyltransferases and cell surface proteins within the 30 gene dataset (Table S8).

We found that 30% of the identified down‐regulated genes encode proteins involved in carbohydrate transport and metabolism, over half of which (*agl4*, *malECG* and *apuB*), are involved in malto‐oligosaccharide metabolism and share the same MalR1/MalR2/MalR3 regulon (Khoroshkin et al., 2016; Lanigan et al., 2019). Furthermore, significant down‐regulation was observed for part of the *eps2* operon (Bbr_0442 – Bbr_0451) representing one half of the bidirectional surface‐associated exopolysaccharide (EPS) biosynthesis gene cluster (Fanning, Hall, Cronin, et al., 2012). In addition, gene *accD* (Bbr_1720), putatively encoding an acetyl Co‐A β chain involved in the metabolism of fatty acids, which was previously identified as a gene involved in *B. breve* UCC2003 biofilm formation due to the reduction in biofilm biomass exhibited by the *accD* mutant strain compared to the WT (Kelly et al., 2020). Three down‐regulated genes (locus‐tags Bbr_0113, Bbr_0114 and Bbr_1888) are predicted to encode extracellular structures, of these, locus‐tag Bbr_0114, encoding a SpaH/EbpB family LPXTG‐anchored major pilin is the most significantly down‐regulated gene with a fc of −15.5 (*p*‐value, 2.35^−117^) compared to the control.

Although a substantial portion (62.2%) of up‐regulated genes encode proteins of unknown function, those which could be functionally classified based on KO groups within *B. breve* UCC2003 specific pathways suggest up‐regulated genes fall within 18 protein families (Figure S5; Table S9). Gene enrichment analyses of the 127 up‐regulated genes using the *B. breve* UCC2003 total gene pool as a background indicates the specific enrichment of ABC transporters, genes encoding biofilm‐related proteins, those involved in the diaminopimelate (DAP) pathway and Tad‐like proteins (Table S10).

DEGs within pathways of genetic information processing are exclusively up‐regulated, containing 12 genes encoding transcription factors, including two MerR family metal sensing transcriptional activators (Bbr_1519 and Bbr_1523) and a number of transcription repressors including the SOS response regulator, LexA, cold shock protein, CspA and WhiB, suggested to function in the regulation of cell division. Two genes encoding XerC site‐specific recombinases (Bbr_1113 and Bbr_1548) are among the most significantly upregulated genes, these proteins are known to be important in the process of chromosomal dimer resolution and promote heritable stability. Furthermore, a number of upregulated genes are involved in redox metabolism, including, Bbr_1717 encoding a Pimeloyl‐ACP methyl ester carboxylesterase involved in the biosynthesis of biotin (vitamin B7), and Bbr_1717 encoding a NAD(P)H‐dependent FMN reductase involved in Riboflavin (vitamin B2) metabolism, in addition to Bbr_1222 encoding a MutT/nudix family phosphohydrolase predicted to function in the removal of deleterious oxidised nucleotide derivatives and Bbr_0205 encoding a predicted polyketide synthase.

Upregulation was observed for the *pel* operon (Bbr_0044 – Bbr_0048), protein products of orthologous genes of Gram‐positive origin have been demonstrated to be responsible for the biosynthesis of Pellicle (Pel) polysaccharides (Whitfield et al., 2020). Furthermore, upregulation was observed for two of the four dihydrodipicolinate synthase‐encoding genes, *dapA3* and *dapA4*, indicated to catalyse the rate‐limiting step within the DAP pathway, responsible for the biosynthesis of bacterial cell wall (Impey et al., 2020). In addition, increased expression was observed of cluster (Bbr_0133 – Bbr_0137) specifying a tight‐adherence (Tad) type IVc pili (T4cP) previously identified as an essential colonisation factor promoting colonic epithelial proliferation of *B. breve* UCC2003 in vivo (O'Connell Motherway et al., 2019).

### Upregulated chromosomal genes in the presence of pMP7017 possess a codon usage profile compatible with the altered cellular tRNA pool due to pMP7017‐expressed tRNAs

Codon usage, an intrinsic feature within ORFs, has recently been documented to influence mRNA levels in fungi and eukaryotes in a translation‐independent manner, revealing the emerging role of codon usage in transcription level regulation (Bahiri‐Elitzur & Tuller, 2021; Newman et al., 2016; Zhao et al., 2021; Zhou et al., 2016). As such, coding sequence composition may also have important implications for gene expression and transcriptional regulation in bacteria. To investigate the possible effect of pMP7017‐encoded tRNAs on gene expression levels in the host, we calculated the average RSCU values for each codon corresponding to pMP7017‐expressed tRNA anti‐codons for up‐ and down‐regulated *B. breve* UCC2003 chromosomal gene sequences and compared these to the RSCU values for the total *B. breve* UCC2003 and pMP7017 transcriptomes. This analysis revealed that upregulated *B. breve* UCC2003 chromosomal genes contain a more congruent RSCU profile with that of the pMP7017 transcriptome and are likely more compatible with the altered tRNA pool, while down‐regulated genes present with a more divergent RSCU profile (Figure S6). Changes in global tRNA abundance due to pMP7017‐expressed tRNAs may therefore enhance the stability of transcripts enriched for these cognate codons, or indeed, alter the expression of specific transcripts through a mechanism of tRNA modulation as indicated for eukaryotes (Bahiri‐Elitzur & Tuller, 2021; Newman et al., 2016; Zhao et al., 2021; Zhou et al., 2016).

### Prevalence, synteny and variability of pMP7017 and related megaplasmids

Screening of pMP7017 genes against the reads of 1846 metagenomic samples across different geographical locations revealed 206 pMP7017‐positive samples with reads mapping to >40% of megaplasmid genes, however only 10 of these samples, including samples from Estonia, Finland, Japan, Russia and the UK, contained mapped pMP7017 genes above the >70% threshold (Table S11). Due to the low abundance and frequency of pMP7017‐homologues, it was only possible to recover and reconstruct a single megaplasmid from the final metagenome‐assembled genomes (Figure S7).

**FIGURE 3:**
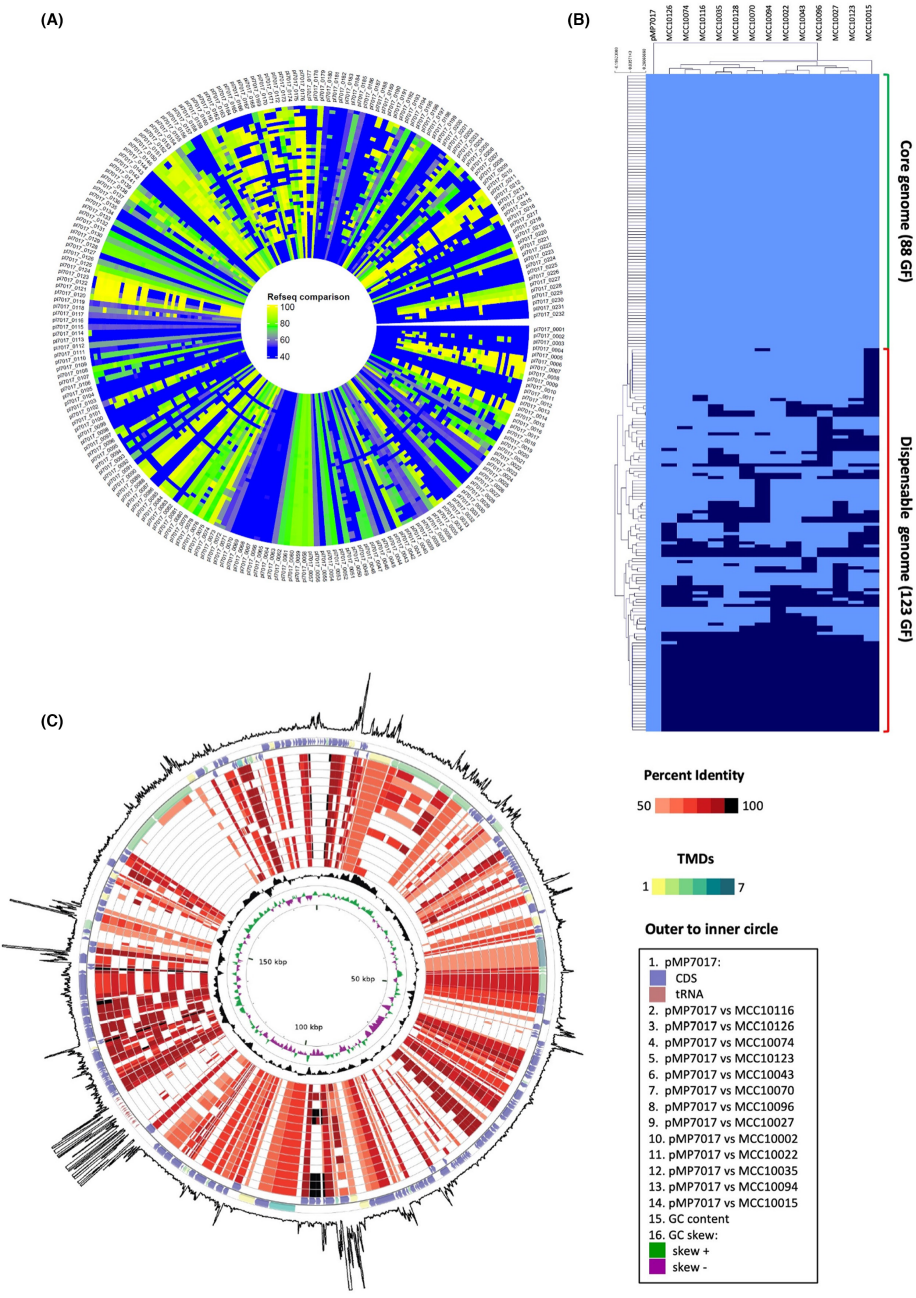
Prevalence, synteny and variability of bifidobacterial megaplasmid genomes. (A) The heatmap presents the percentage of sequence identity of each pMP7017 protein (locus tags given in outer ring) against those of the 43 *Bifidobacterium longum* pMP7017 positive Refseq strains presenting with 0.0001 *E*‐value cut‐off, 50% of sequence coverage and 40% identity cut‐off. The colour ranges from blue to yellow with increasing identity. (B) Hierarchical clustering heatmap representing the variability of pMP7017 family plasmids in terms of presence/absence of gene families computed using the all‐versus‐all BlastP algorithm and MCL clustering. The number of core (those present in all genomes) and dispensable (those present in some genomes) gene families are indicated. (C) Circular genome atlas of pMP7017 with mapped orthologs (defined as reciprocal best BlastP hits with >50% identity over >80% of both protein lengths) in 14 publicly available *B. longum* MCC strain genomes. The outermost circle displays RNAseq‐mapped reads, while the remaining circles are as outlined in the legend. pMP7017 coding sequence (CDS) are colour‐coded by the number of predicted transmembrane domains (TMDs).

We then focused on the experimentally verified replicase (B7017_p0146) and searched for this protein against all available *Bifidobacterium* genomes in the Refseq database (Dineen et al., 2021). Genomes which contained a homologue of the pMP7017 replicase protein were then considered pMP7017‐positive once all backbone functions, including proteins involved in conjugation and partitioning, were present within the following parameters (0.0001 *E*‐value cut‐off, 70% of sequence coverage and 70% identity cut‐off). Our analysis revealed that 43 out of 375 assessed *B. longum* strains are positive for all pMP7017 backbone proteins, thus are assumed to contain a pMP7017‐related megaplasmid or integrated element (Figure 3A). In addition, a single pMP7017‐positive *B. breve* strain was identified from this analysis, *B. breve* MCC1454 isolated from infant faeces (Biosample: SAMN03978804). However, *B. breve* MCC1454 shares an average nucleotide identity (ANI) of >99.95% with the pMP7017 host genome *B. breve* JCM7017. As this value is above the accepted threshold for species differentiation (≥95% ANI) these genomes thus constitute two closely related strains, representing a positive control for the presence of pMP7017 within *B. breve* in the natural environment. Collectively, this data supports the supposition that *B. longum* is likely the natural host of this plasmid family due to the presence of this megaplasmid in ~11.5% of *B. longum* strains compared to ~0.19% of the 537 *B. breve* strains analysed (Odamaki et al., 2018).

To assess the diversity of gene carriage among bifidobacterial megaplasmids, 14 *B. longum* genomes previously demonstrated to contain a pMP7017‐related megaplasmid were compared (Figure 3C) (Odamaki et al., 2018). This comparative analysis revealed the conservation of 88 Markov clustering (MCL) families representing the core genome and includes genes specifying replication and partition functions, in addition to those encoding type four secretion system components (Figure 3B). A total of 123 MCL families, representing 53% of the pMP7017 predicted coding sequences were shared by some but not all strains, thus constituting the dispensable genome. Furthermore, 28 MCL families are unique to pMP7017 and mainly represent functionally unassigned genes.

Further to this analysis, using the established pMP7017 replicase‐coding sequence and iteron‐containing region as a bait we were able to identify four additional and recently sequenced putative pMP7017 homologues in the NCBI database. When comparing these whole genome sequences to the pMP7017 reference genome, we observed varying degrees of sequence conservation, in addition to a number of frameshifts representing deletions or insertions among sequences and a large inversion (nt coordinates, 84,894‐128,826) encompassing genes B7017_p0114 – B7017_p0153 inclusive was observed within the pMP7017 genome (Figure S8). These data indicate that the bifidobacterial megaplasmids so far identified in bifidobacterial genomes are structurally related and thus share a common ancestor. However, the diversity among accessory gene cargos as well as divergence between orthologous protein sequences suggest that these megaplasmids have experienced extensive independent evolutionary pressures.

## DISCUSSION

In this work, the megaplasmid pMP7017 was transcriptionally profiled, while the transcription termination sites of active transcriptional units were mapped. Using this dataset, we were able to identify a consensus promoter typical of bifidobacteria with conserved hexamers TATAAT (−10 region) and TTGACA (−35 region), while degeneration of the promoter consensus motif is observed for genes with lower levels of transcription. In addition, the most prevalent spacer length among the deduced promoters of highly expressed genes was that of 17 bps, with a ± 1 bp deviation in spacer length observed within moderate strength promoter sequences. Together this data suggests the AT% of the −10 hexamer in addition to the length of the spacer region appears to highly impact gene transcription levels, consistent with what has been observed in the transcriptional profiling and promoter mapping of the *B. breve* UCC2003 genome (Bottacini et al., 2017). As homologues of a large number of our identified genes are present among pMP7017‐related plasmids, this dataset constitutes a transcriptomic reference which may be implemented for future investigations of gene expression in members of this plasmid family.

A primary motivation of systems biology research is to understand gene functions and their interactions within an overarching community or global context. Given the rapid increase in next‐generation sequencing data and more comprehensive databases, updating structural and functional annotations of genes as well as deciphering transcriptional profiles, are essential components in building a foundation towards this understanding. Comprehensive computational analysis of each pMP7017 coding sequence enabled the assignment of putative functional roles to 15 genes (6.6% of the pMP7017 predicted genome) and further refine the functional annotation of 21 transcriptionally active genes (9.2% of the pMP7017 predicted genome). However, 51% of transcriptionally active pMP7017 genes remain functionally unassigned; this value is substantially higher than that of the estimated 26% and 22.5% representing the average percentage of hypothetical genes among all available *B. breve* and *B. longum* genome sequences, respectively (Albert et al., 2019; Bottacini et al., 2014). Thus, considering the cost of expression to the host cell it is possible that these genes encode functionally important proteins.

Horizontal transfer of plasmid genes enables the adaptation of bacteria to diverse environmental conditions, facilitating their evolution. However, non‐self‐genomes impose multi‐level pressures on the host chromosome with large mobile elements, such as megaplasmids, depleting cellular resources for their maintenance, horizontal spread and expression, resulting in a reduction of host fitness. While compensatory evolution can lessen these fitness costs over time, the initial cost(s) associated with plasmid carriage may represent a constraint hindering horizontal and vertical transmission of these genetic elements, thereby reducing their chances of survival in bacterial communities. These biological conflicts are widespread among bacteria, as a result, extensive conflict‐related adaptations have evolved such as CRISPR/*cas* systems and RM systems in the host, as well as plasmid‐encoded TA systems and anti‐restriction proteins. In this work, an integrated approach of transcriptome analysis and functional characterisation of pMP7017‐encoded proteins revealed transcriptional activity of both offensive and defensive adaptions, including four Type II TA systems, an anti‐restriction protein and a presumed Type IIL RM system. While experimental validation of their functionality is required, pMP7017‐encoded TA systems likely ensure maintenance and stability of the plasmid in the host, while methylases and/or anti‐restriction proteins enhance the success of its horizontal transmission by preventing restriction of plasmid DNA upon entry into a recipient cell.

Furthermore, our analysis enabled the identification of a highly transcribed tmRNA housekeeping sRNA, this unique RNA facilitates the rescue of stalled ribosomes and promotes the degradation of defective mRNAs. Prior to this work, the smallest megaplasmid known to carry a tmRNA was that of the 567.3 kb megaplasmid, pCPAA3, isolated from *Curtobacterium pusillum* AA, as such, pMP7017 now represents the smallest plasmid known from literature and the first plasmid of the *Bifidobacterium* genus harbouring a tmRNA. Although the contribution of tmRNA^pMP7017^ to the biology of the megaplasmid and its interaction with the host‐encoded trans‐translational system remains to be uncovered, tmRNA^pMP7017^ likely aids the host in mitigating toxicity effects resulting from the accumulation of truncated polypeptides and the depletion of translational ribosomes. Equally, pMP7017 may benefit from the unique functional aspects of the specific tmRNA variant it encodes or profit from the increased gene dosage.

While pMP7017 expresses a large number of tRNAs, a characteristic which has been widely suggested to confer benefits to the host, our analyses suggest that pMP7017‐carriage of tRNA genes may in fact facilitate adaption of the cellular tRNA pool to the codon usage of pMP7017‐encoded genes. Interestingly, the codon usage profile of highly transcribed pMP7017 genes was established to be more similar to that of the *B. longum* subsp. *longum* genome rather than that of the host bacterium, *B. breve* JCM7017, from which it was originally derived. As both *B. breve* and *B. longum* subsp. *longum* represent common inhabitants of the infant gut, in addition to *the* co‐occurrence of known pMP7017‐homologues in both species, these data may support the supposition that an ancestral plasmid of pMP7017 transferred to *B. breve* JCM7017 from a *B. longum* subsp. *longum donor*. Strain‐specific evolution and domestication of pMP7017 may thus have resulted in its diversification. This is reflected in the observed divergence between orthologous protein sequences and a high frequency of genomic reorganisation, represented by frameshifts and inversions within the pMP7017 genome relative to pMP7017‐related megaplasmids of *B. longum* subsp. *longum* origin. Furthermore, considering the prevalence, albeit low, of pMP7017‐related megaplasmids within this specific bacterial lineage, deeper analysis of variations among more distantly related plasmids would serve in the understanding of the evolution of this plasmid family, revealing the impact(s) of structural variations on gene expression and potentially unveiling strain‐specific adaptations.

While no clear discernible pattern was observed between transcriptionally affected chromosomal genes, it is evident that specific gene clusters and genes encoding functionally comparable proteins were similarly affected, together representing 8% of the *B. breve* UCC2003 genome. Although the biological significance of this is not yet apparent, it is possible that these changes may be consequential to the altered expression of chromosomal regulators, as observed for the 21 kb pathogenicity island (PAI) of *Rhodococcus equi* (Coulson et al., 2015).

It is well established that various polysaccharides have distinct physical properties and functional roles during biofilm formation, affecting both spatial organisation and integration. *Bifidobacterium breve* UCC2003 has been demonstrated to contain a large EPS cluster containing two bidirectional EPS operons, *eps1* and *eps2*, specifying two alternative EPSs (Fanning, Hall, Cronin, et al., 2012). In our analysis, a down‐regulation of the *eps2* operon in the presence of pMP7017 was observed with no statistically significant differential expression observed at the *eps1* operon. Furthermore, our data indicates an upregulation of a presumed *pel* polysaccharide biosynthesis cluster (Fanning, Hall, & van Sinderen, 2012). Pel polysaccharide, is a thin polysaccharide that is tightly associated with the peptidoglycan layer which has been shown to be essential for the formation of mixed integrated biofilm communities in *Pseudomonas aeruginosa* and *S. aureus* co‐cultures, while a reduction of 20–40 μm in microcolony height was observed with a *P. aeruginosa* PAO1 *pel* mutant compared to the WT, where the authors propose that the absence of Pel may result in stiffer, more rigid biofilms (Chew et al., 2014; Periasamy et al., 2015). Altered expression of host chromosomal genes involved in the formation of surface polysaccharides and adhesion‐related functions involved in biofilm production have been reported for a number of bacteria upon acquisition of conjugative plasmids, which favour plasmid transfer in these spatially structured populations (Ghigo, 2001; Lang & Johnson, 2015; Matsumoto et al., 1998; Parashar et al., 2013; Weber et al., 2015). Considering this, it is tempting to speculate that these findings indicate dynamic remodelling of the *B. breve* UCC2003 cell envelope composition which could indirectly enhance horizontal transmission of pMP7017, resulting from the rheological contributions of these EPSs.

In‐depth analysis of gene expression profiles which differentiate experimental groups is critical in the discovery and analysis of potential transcriptional networks between chromosomal and plasmid genes and to identify key factors which may contribute to the maintenance of pMP7017 in natural populations. Transcriptome comparison of pMP7017‐free and pMP7017‐containing hosts with more divergent genotypes may refine the dataset and enable the identification of host‐specific and/or general impacts resulting from plasmid carriage, as observed for pCAR1 with three distinct *Pseudomonas* host strains and F‐plasmid in two different *Escherichia coli* strains (Harr & Schlötterer, 2006; Shintani et al., 2010). In addition, investigation of the effects of plasmid carriage on the host cell may elucidate the mechanisms underpinning the acquisition of horizontally acquired genes and expose how pMP7017 and related plasmids adapt to new host cell backgrounds in the natural environment.

## AUTHOR CONTRIBUTIONS

**Rebecca L. Dineen:** Conceptualization (lead); formal analysis (lead); investigation (lead); methodology (lead); project administration (lead); visualization (lead); writing – original draft (lead). **Francesca Bottacini:** Investigation (supporting); validation (equal); writing – review and editing (supporting). **Mary O'Connell‐Motherway:** Funding acquisition (equal); writing – review and editing (supporting). **Douwe van Sinderen:** Conceptualization (supporting); funding acquisition (equal); supervision (lead); validation (equal); writing – review and editing (lead).

## FUNDING INFORMATION

No funding information provided.

## CONFLICT OF INTEREST STATEMENT

Authors have no conflict of interest to declare.

## Supporting information


Data S1:
