# Supplementary material for: Transcriptional landscape of the pMP7017 megaplasmid and its impact on the Bifidobacterium breve UCC2003 transcriptome
Source: Microb Biotechnol. 2024 Jan 11;17(1):e14405. doi: 10.1111/1751-7915.14405 (PMC10832533; doi:10.1111/1751-7915.14405)
Supplement: Supplementary file 1 — Data S1: [file MBT2-17-e14405-s001.docx]

## SUPPLEMENTAL MATERIAL

##### **
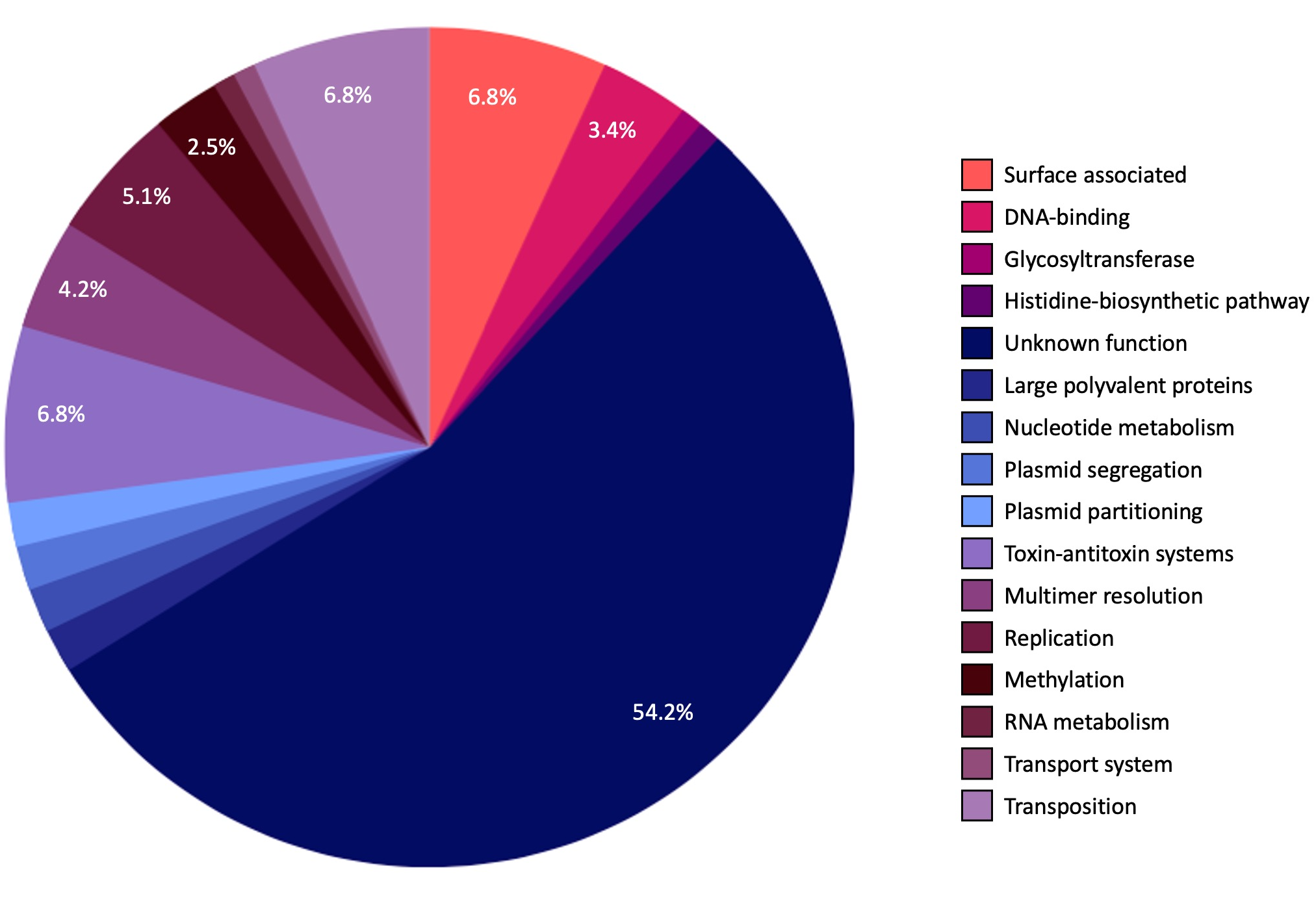
**

Figure S1. Pie chart representation of transcriptionally active predicted pMP7017-genes, grouped by functional categories

##### Categories within in the legend are presented as ordered in the pie chart clockwise from the 12 o’clock position.


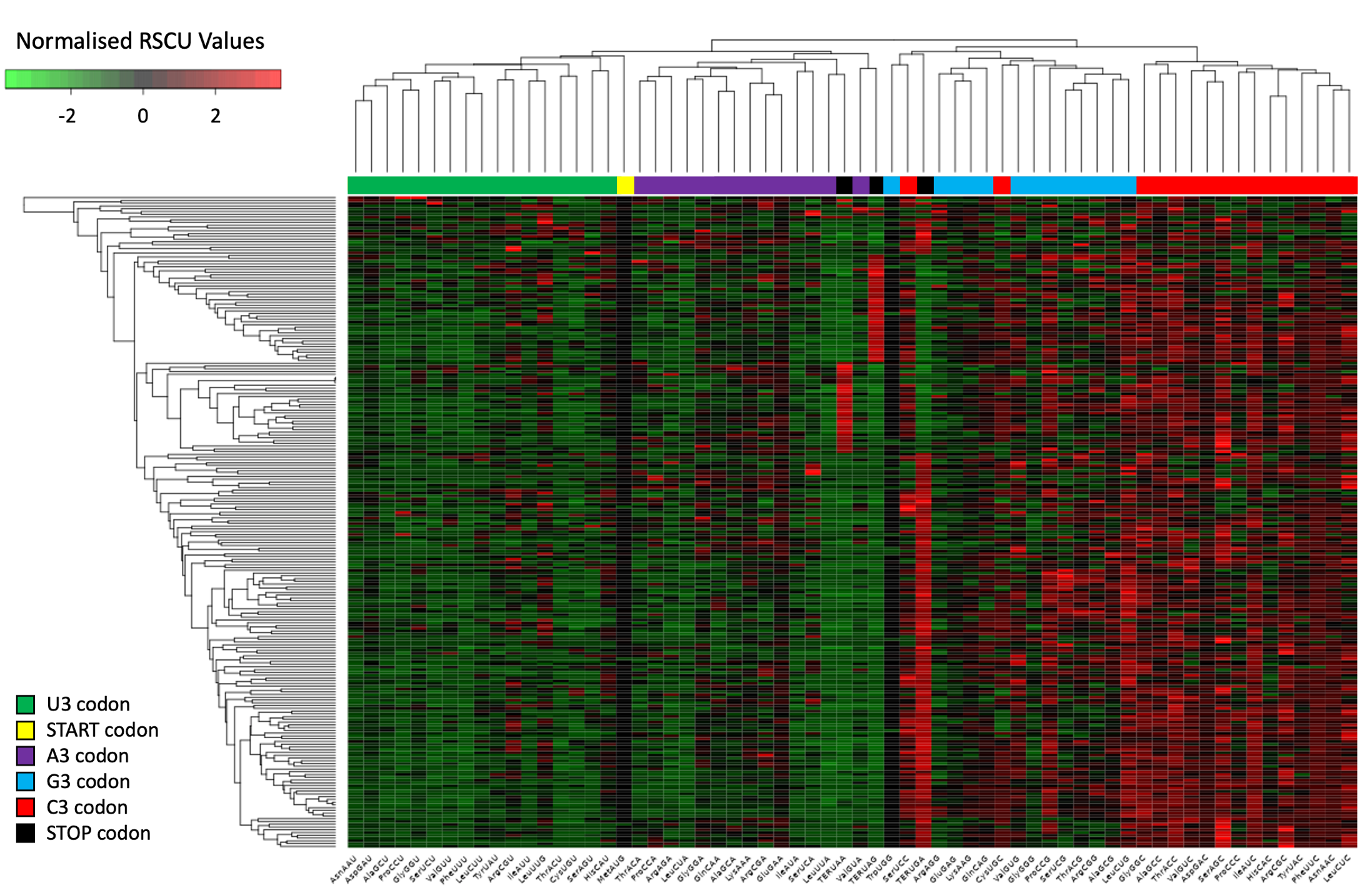


Figure S2. Codon usage bias of individual genes within the pMP7017 genome based on RSCU values

##### The two dimensional cluster heatmap is based on the frequency of codons for each putative gene within the pMP7017 genome. Genes are in rows and codons are in columns (colour coded by synonymous third codon position - top). Codons corresponding to methionine (yellow), tryptophan and stop codons (black) are included. Colour intensity changes from green to bright red with an increase in frequency of a particular codon.


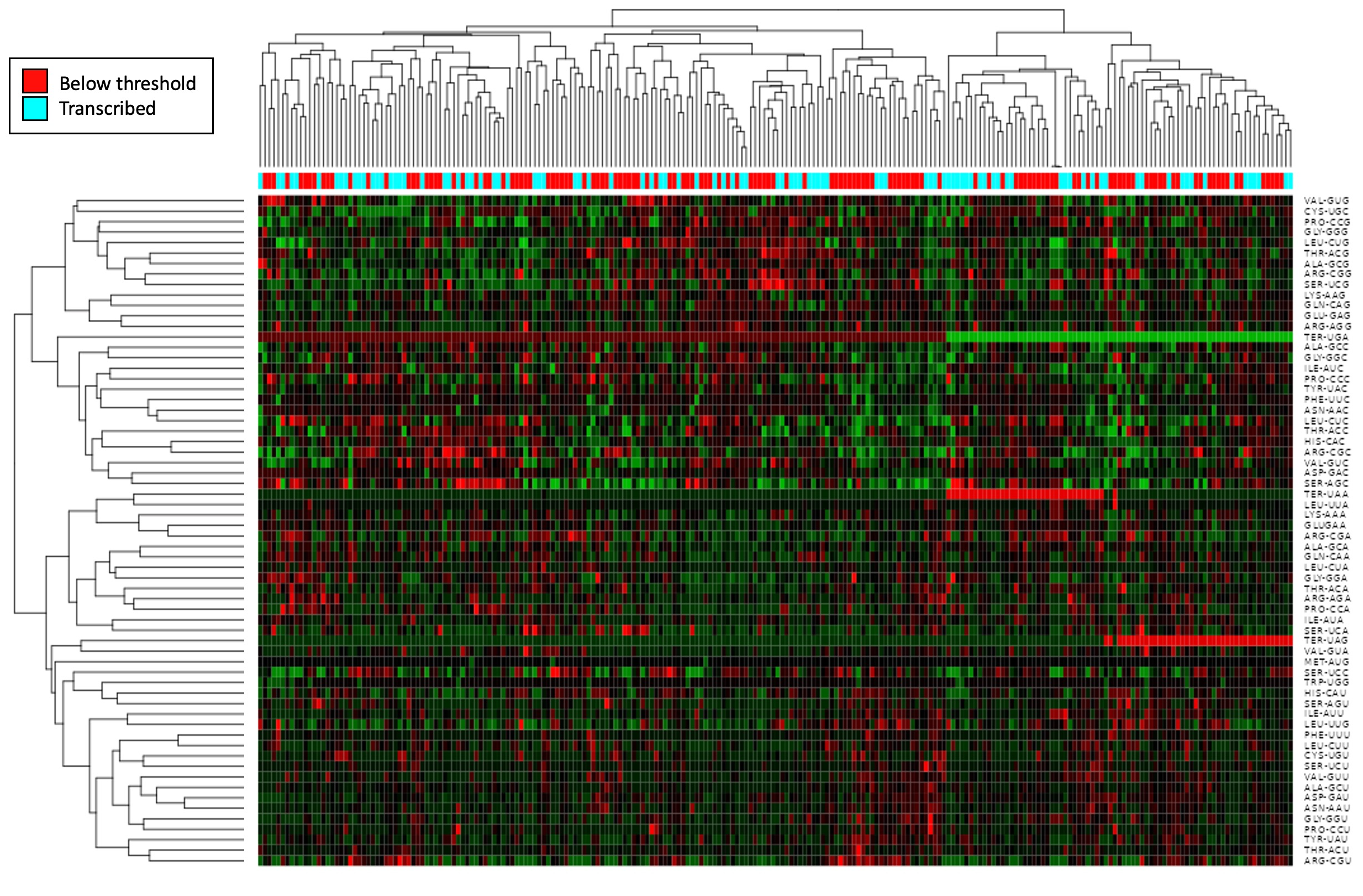


Figure S3. Codon usage comparison of pMP7017-encoded transcribed genes vs non-transcribed genes at mid-log growth of *B. breve* UCC2003

##### Codon usage based two dimensional cluster heatmap. Heatmap based on the usage (frequency) of codons for the pMP7017 genome. Colour intensity changes from bright red to green with an increase in frequency of a particular codon. Genes transcribed at mid-log growth of *B. breve* UCC2003 are indicated in cyan, while non-transcribed genes (below-threshold) are indicated in red (top).


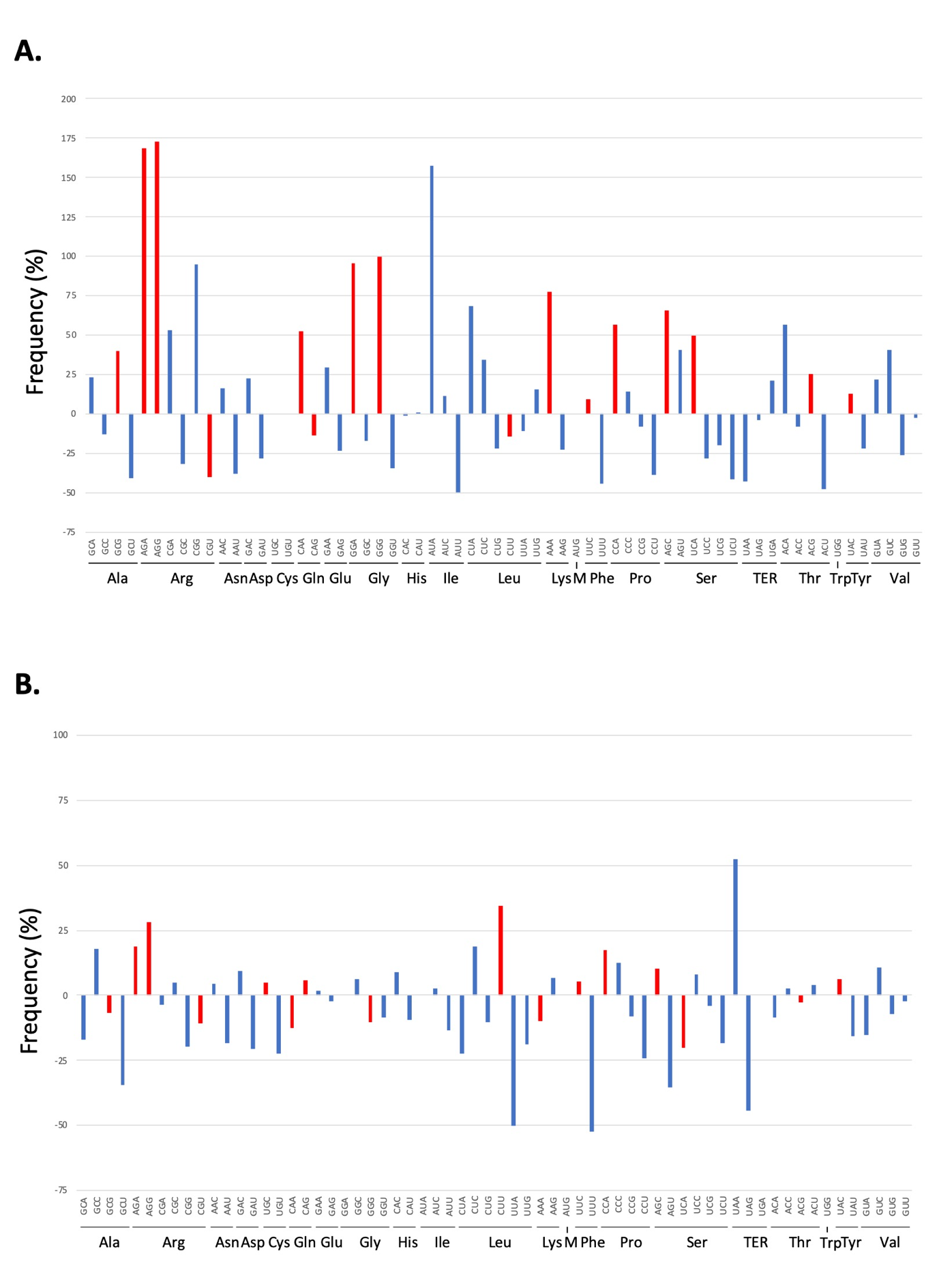


Figure S4. Codon usage frequencies of pMP7017 genes vs JCM7017: tRNAs and codon usage

##### **A.** Bar height is based on the codon usage frequency of pMP7017 genes divided by the codon usage frequency of *B. breve* JCM7017. Red bars correspond to pMP7017-expressed tRNA anti-codons, while blue bars represent all other tRNA anti-codons. **B.** Bar height is based on the codon usage frequencies of highly transcribed pMP7017 genes divided by the codon usage frequencies of the total pMP7017 genome.

#####
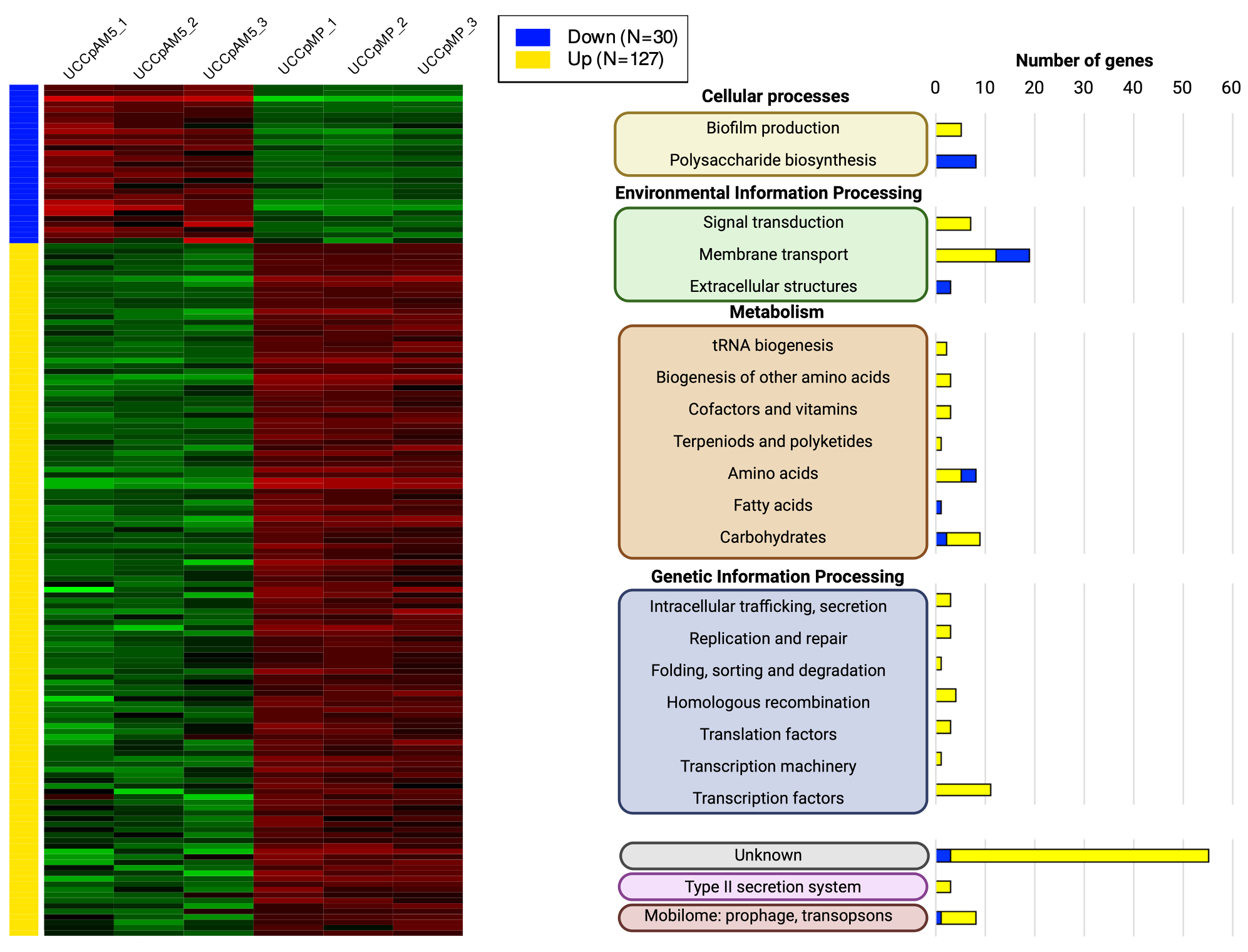


Figure S5. Differential expression of *B breve* UCC2003 chromosomal genes in the presence of pMP7017

##### Heat map representing the log2 transformed expression values of *B. breve* UCC2003 chromosomal genes with altered levels of transcription in the presence of pMP7017 at mid-logarithmic growth. Samples UCCpAM5_1-3, represent replicates 1-3 of *B. breve* UCC2003 containing pAM5 (pAM5 is included to control for any transcriptional effects due to the use of antibiotic), while samples UCCpMP_1-3, represent replicates 1-3 of *B. breve* UCC2003 harbouring pMP7017 (left). COG category assignments of differentially expressed genes, grouped by protein families is presented to the left of the chart. The number of DEGs within each protein family is given, down-regulated genes are indicated by blue bars and up-regulated genes are represented by yellow bars. Detailed analysis of each DEG are presented in (Table 6 and Table 8).


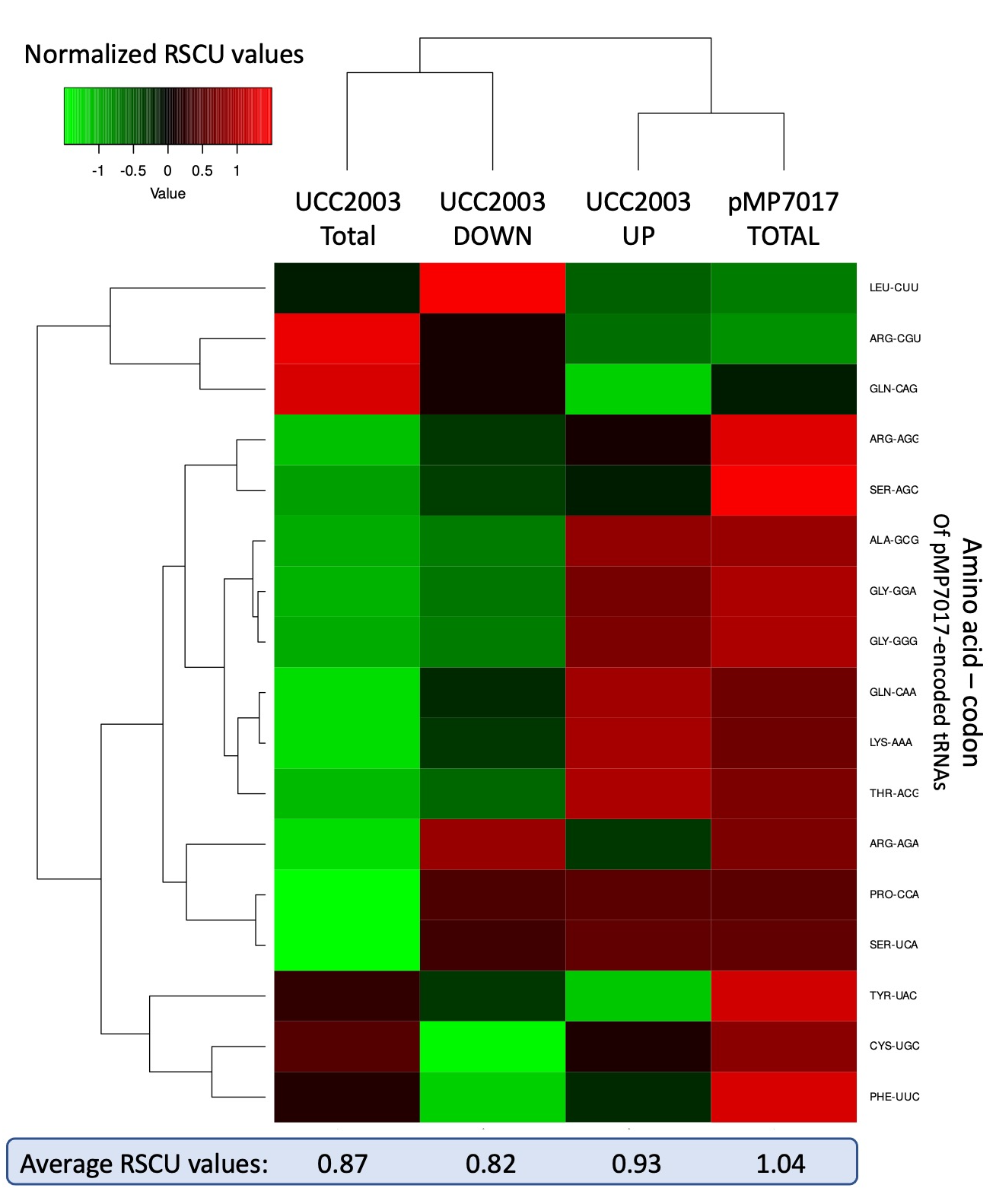


Figure S6. Codon usage comparison of chromosomal DEGs in the presence of pMP7017 and its relationship with pMP7017 encoded tRNA anti-codons

##### The two-dimensional cluster heatmap comparing the frequencies of codons for all coding sequences within the *B. breve* UCC2003 genome, chromosomal DEGs and the pMP7017 genome. Gene datasets are in columns and codons corresponding to pMP7017-expressed tRNA anti-codons are in rows*.* Colour intensity changes from green to bright red with an increase in frequency of a particular codon.


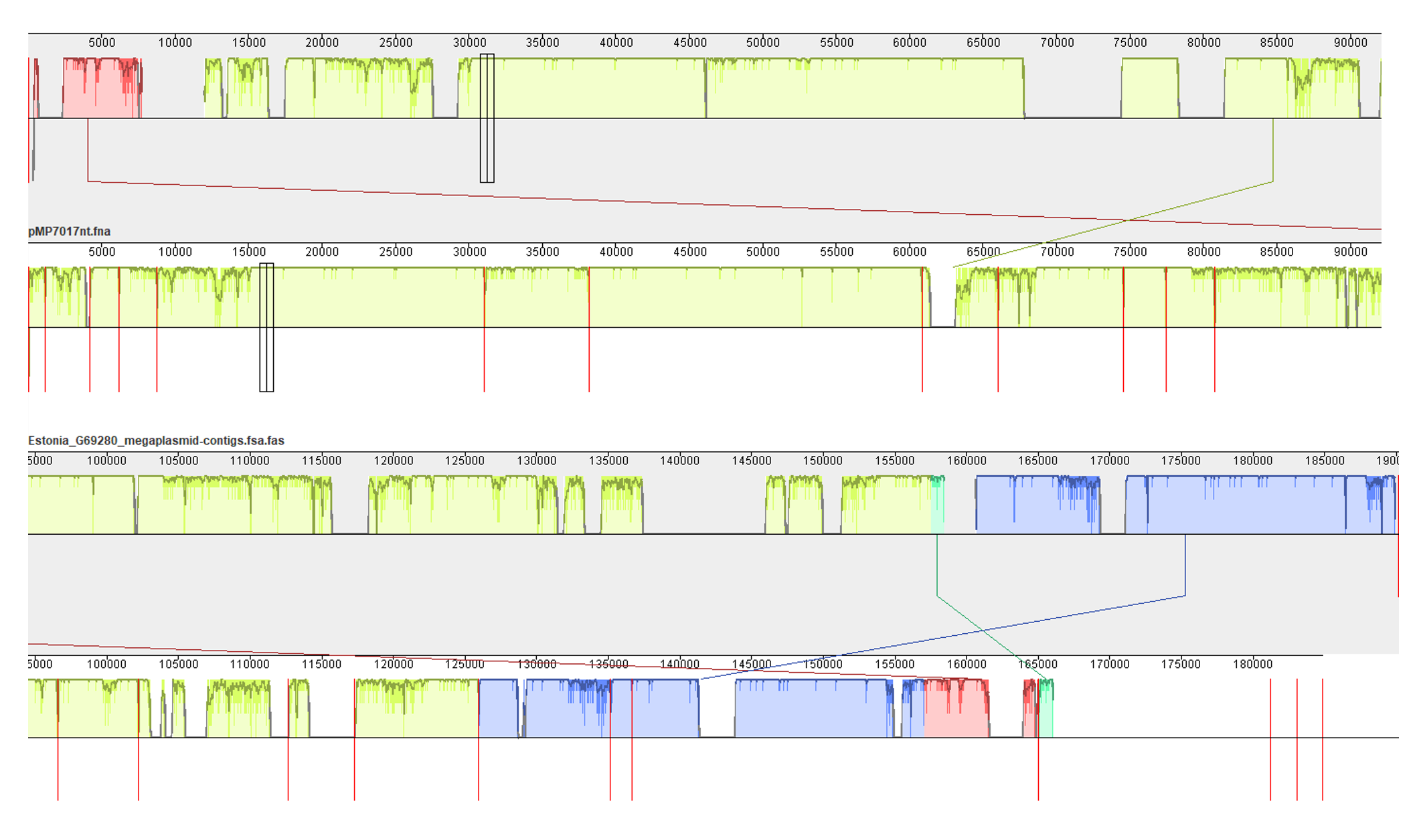


Figure S7. MAP construction of pMP7017 from metagenomic sample

##### MAUVE alignment of an assembled pMP7017-positive metagenomic sample from Estonia and pMP7017. Local collinear blocks within each aligned sequence are coloured to indicate syntenic regions, while the histogram within each box represent the degree of sequence similarity.


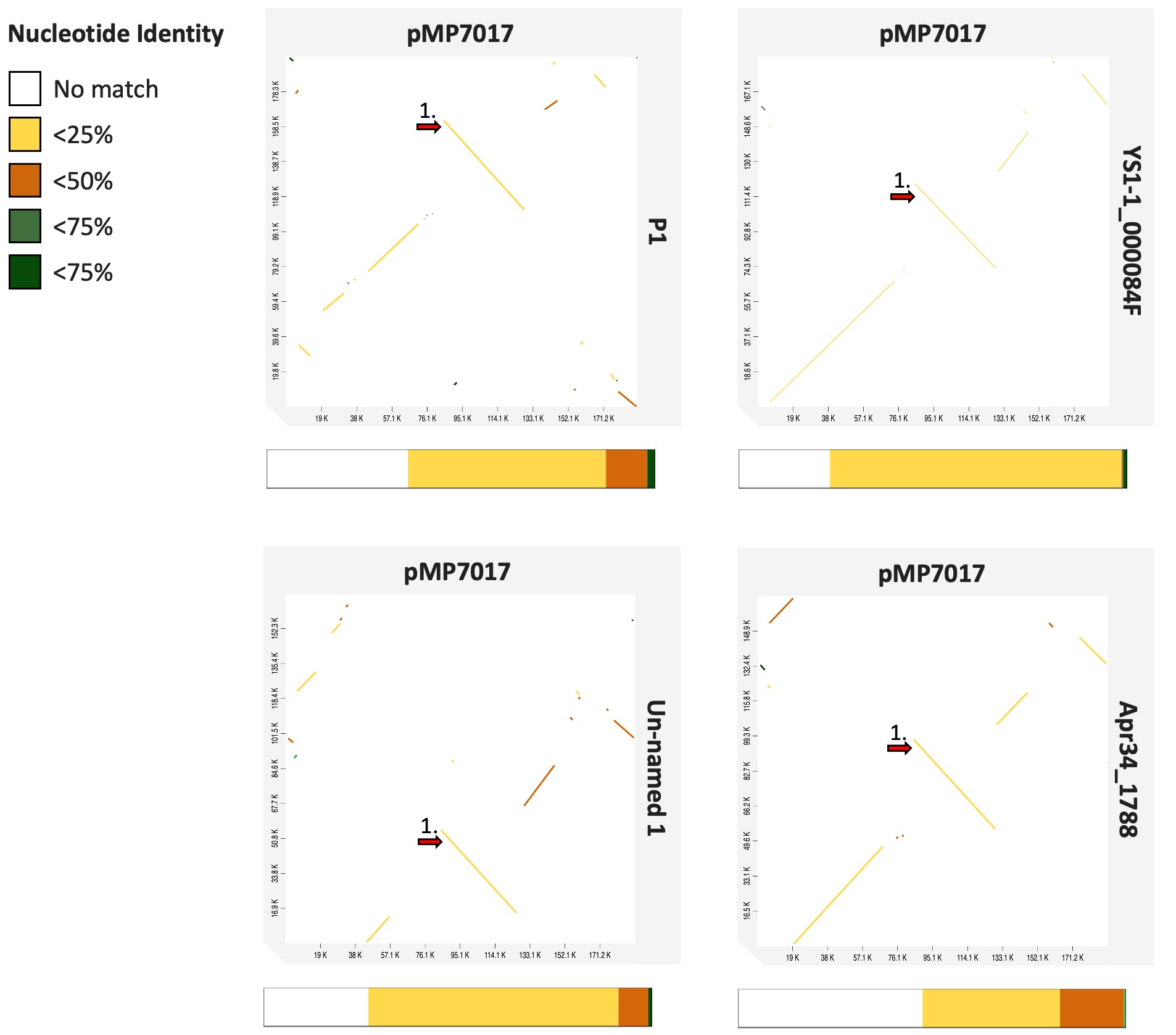


Figure S8. Genome synteny and co-linearity of pMP7017 family plasmids

##### Dot-plot comparison showing conserved and inverted regions found in pMP7017-homologs relative to the pMP7017 genome. The plots highlight a large inversion (red arrow, numbered (1.)), corresponding to nucleotide coordinates; 84 894-128 826, within the pMP7017 genome.


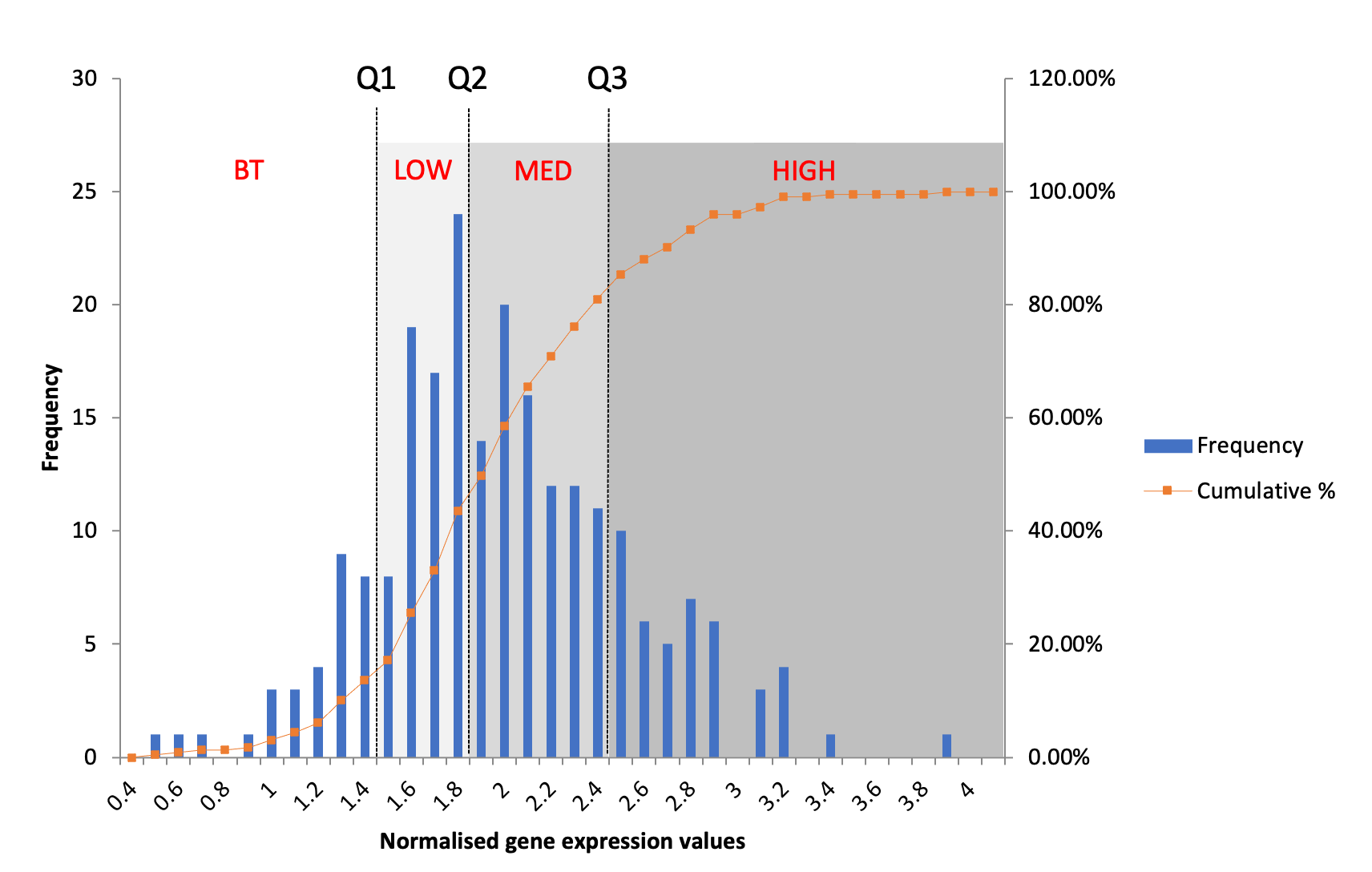


Figure S9. Establishing gene expression thresholds

The chart shows the quantile thresholds [Q1, Q2 and Q3] (indicated with dashed lines) on the normalized gene expression levels (RNA-seq) from pMP7017. Genes above the upper threshold are classified as highly expressed genes, whereas genes below the lower threshold are classified as not expressed (BT = Below Threshold). The data refers to protein coding genes only and does not include tRNA gene transcripts.

Table S1. Functional assignment of proteins within the presumed pMP7017-specific regulon based on KEGG pathways and COG functions

|  | KEGG | | COG | |
| --- | --- | --- | --- | --- |
| Locus-tag | E-value | Accession | E-value | Accession |
| B7017_p0032 | - | - | - | - |
| B7017_p0044 | - | - | - | - |
| B7017_p0046 | 1.82E-10 | K12019 | - | - |
| B7017_p0067 | - | - | - | - |
| B7017_p0068 | - | - | - | - |
| B7017_p0081 | - | - | - | - |
| B7017_p0082 | - | - | - | - |
| B7017_p0083 | 2.87E-38 | K19126 | 1.06E-31 | COG5934 |
| B7017_p0084 | 1.59E-46 | K19123 | 2.85E-44 | CD09669 |
| B7017_p0085 | 1.75E-04 | K19046 | 6.78E-05 | COG5949 |
| B7017_p0086 | - | - | - | - |
| B7017_p0087 | 2.33E-57 | K03720 | - | - |
| B7017_p0088 | 7.96E-75 | K19124 | 7.96E-75 | COG1857 |
| B7017_p0089 | 4.92E-41 | K19125 | 4.92E-41 | COG1688 |
| B7017_p0090 | 6.05E-98 | K15342 | 6.05E-98 | COG1518 |
| B7017_p0091 | 2.36E-15 | K09951 | 2.73E-11 | COG1343 |
| B7017_p0092 | - | - | - | - |
| B7017_p0093 | - | - | - | - |
| B7017_p0094 | 3.14E-11 | K18955 | 3.14E-11 | COG4008 |
| B7017_p0095 | - | - | - | - |
| B7017_p0096 |  | na | 2.28E-61 | NOVA0UKS |
| B7017_p0097 |  | na | 1.63E-12 | COG0326 |
| B7017_p0098 | 1.63E-12 | K06400 |  | COG1961 |
| B7017_p0103 | 2.54E-76 | K03709 | - | - |
| B7017_p0104 | 2.32E-34 | K18917 | 1.19E-35 | NOVA81QL |
| B7017_p0105 | - | - | - | - |
| B7017_p0106 | 8.52E-19 | K05844 |  |  |
| B7017_p0107 | - | - | - | - |
| B7017_p0108 | 3.10E-55 | K02342 | 3.10E-55 | COG2176 |
| B7017_p0134 | 1.79E-05 | K04074 | 1.79E-05 | COG3264 |
| B7017_p0149 | - | - | - | - |
| B7017_p0150 | - | - | - | - |
| B7017_p0151 | 1.81E-21 | K03528 | 9.60E-59 | COG3115 |
| B7017_p0152 | 8.54E-113 | K04001 | 8.54E-113 | COG1514 |
| B7017_p0153 | 2.06E-28 | K01091 | - | - |
| B7017_p0154 | - | - | - | - |
| B7017_p0155 | - | - | - | - |
| B7017_p0156 | 1.50E-20 | K22463 | 1.50E-20 | COG4933 |
| B7017_p0157 | - | - | - | - |
| B7017_p0158 | - | - | - | - |
| B7017_p0159 | - | - | - | - |
| B7017_p0181 | - | - | - | - |
| B7017_p0182 | 3.62E-40 | K00127 | - | - |
| B7017_p0183 | 4.85E-28 | K26115 | - | - |
| B7017_p0184 | 1.62E-31 | K07284 | 1.62E-31 | COG3764 |
| B7017_p0197 | 5.39E-89 | K00571 | 5.39E-89 | COG0863 |
| B7017_p0198 | 2.28E-27 | K03111 | 2.28E-27 | COG0629 |
| B7017_p0215 | - | - | - | - |
| B7017_p0216 | - | - | - | - |
| B7017_p0217 | - | - | - | - |
| B7017_p0218 | - | - | - | - |
| B7017_p0219 | - | - | - | - |
| B7017_p0220 | 6.50E-38 | K02955 |  |  |
| B7017_p0221 | - | - | - | - |
| B7017_p0222 | 1.92E-32 | K07313 |  |  |
| B7017_p0223 | - | - | - | - |

Table S2. Updated annotation of transcriptionally active genes

| Locus_tag | Accession | Current annotation | Proposed annotation | Software | Accession | Description | Confidence (%) | Probability (%) | E-value |
| --- | --- | --- | --- | --- | --- | --- | --- | --- | --- |
| B7017_p0014 | AIW55067 | hypothetical protein with a helix-turn-helix motif | HigA | Phyre2 | c6ltyB | Putative antitoxin higa3 | 99.6 | - | - |
|  |  |  |  | HHpred | 7EWC-C | Antitoxin HigA2 | - | 98.62 | 3.80E-06 |
|  |  |  |  | Pfam | HTH_37 | Helix-turn-helix domain | - | - | 3.30E-12 |
| B7017_p0015 | AIW55068 | toxin-antitoxin system | HigB | Phyre2 | c6af3-C | HigB toxin | 99.9 | - | - |
|  |  |  |  | HHpred | 6AF4_C | HigB toxin | - | 99.62 | 2.90E-14 |
|  |  |  |  | Pfam | Gp49 | Phage derived protein Gp49-like (DUF891) | - | - | 20E-25 |
| B7017_p0019 | AIW55072 | antidote protein, toxin-antitoxin system | XRE-family transcriptional regulator | Phyre2 | c3trb-A | antidote protein HigA | 99.9 | - | - |
|  |  |  |  | HHpred | 6HU8_A | Transcriptional regulator ComR | - | 98.69 | 2.50E-07 |
|  |  |  |  | Pfam | HTH_3 | Helix-turn-helix 3 | - | - | 6.00E-08 |
|  |  |  |  | CDD | cl22854 | HTH_XRE super family |  |  | 4.43E-23 |
| B7017_p0020 | AIW55073 | cell surface protein, cysteine-rich extracellular domain | PrgA-like protein | Phyre2 | c7kog-B | Myosin heavy chain isoform mhc_x1 | 91.1 | - | - |
|  |  |  |  | HHpred | 6Z9L-A | PrgA | - | 100 | 9.30E-45 |
|  |  |  |  | Pfam | CAP | Cysteine-rich secretory protein family | - | - | 6.80E-07 |
| B7017_p0021 | AIW55074 | Sortase family protein | Pilin specific class C sortase | Phyre2 | c2xwg- | Sortase class C | 100 | - | - |
|  |  |  |  | HHpred | 7CG9_A | Class C sortase | - | 99.95 | 4.20E-25 |
|  |  |  |  | Pfam | Sortase | Sortase domain | - | - | 1.20E-21 |
| B7017_p0129 | AIW55183 | DNA-binding helix-turn-helix protein HipB | XRE family transcriptional regulator | Phyre2 | c2kpj-A | LexA, sos response regulator | 93.5 | - | - |
|  |  |  |  | HHpred | 3PXP_A | MmyB-like transcription regulator MltR, antibiotic | - | 98.29 | 1.40E-05 |
|  |  |  |  | Pfam | HTH_19 | Helix-turn-helix domain | - | - | 2.60E-05 |
|  |  |  |  | CDD | COG3655 | YozG; DNA-binding transcriptional regulator, XRE family |  |  | 2.70E-05 |
| B7017_p0142 | AIW55195 | Putative restriction enzyme | MmeI-like, N-terminal domain | Phyre2 | c5hr4-J | MmeI, DNA-methyltransferase domain | 100 | - | - |
|  |  |  |  | HHpred | 5HR4_J | MmeI | - | 99.96 | 3.70E-27 |
|  |  |  |  | Pfam | MmeI_hel | MmeI, helicase spacer domain | - | - | 8.10E-37 |
|  |  |  |  | Pfam | MmeI_N | MmeI, N-terminal domain | - | - | 3.70E-28 |
| B7017_p0143 | AIW55196 | putative DNA methyl-transferase | MmeI-like, DNA-methyl-transferase domain | Phyre2 | c5hr4-J | MmeI | 100 | - | - |
|  |  |  |  | HHpred | 5HR4_J | MmeI | - | 100 | 2.40E-33 |
|  |  |  |  | Pfam | MmeI_Mtase | MmeI, DNA-methyltransferase domain | - | - | 4.20E-82 |
|  |  |  |  | Pfam | MmeI_TRD | MmeI, target recognition domain | - | - | 7.40E-19 |
| B7017_p0144 | AIW55197 | Methyl-transferase domain protein | MmeI-like, target recognition domain | Phyre2 | c5hr4-J | MmeI | 100 | - | - |
|  |  |  |  | HHpred | 5HR4_J | MmeI | - | 99.56 | 1.00E-12 |
|  |  |  |  | Pfam | MmeI_TRD | MmeI, target recognition domain | - | - | 2.10E-28 |
| B7017_p0170 | AIW55219 | ATPase, AAA superfamily | RecF | Phyre2 | c6wg3-B | Structural maintenance of chromosomes 3 | 99.9 | - | - |
|  |  |  |  | HHpred | 4I99_B | Chromosome partition protein SMC protein | - | 99.75 | 8.40E-17 |
|  |  |  |  | Pfam | AAA_21 | AAA_21 super family | - | - | 5.89E-13 |
|  |  |  |  | CDD | COG1195 | Recombinational DNA repair ATPase RecF |  |  | 1.80E-12 |
| B7017_p0173 | AIW55222 | hypothetical protein | DUF3841 domain protein | Pfam | DUF3841 | Domain of unknown function (DUF3841) | - | - | 1.80E-38 |
| B7017_p0174 | AIW55223 | DNA methylase | MmeI-like RM | Phyre2 | c5hr4-J | MmeI | 100 | - | - |
|  |  |  |  | HHpred | 5HR4_J | MmeI | - | 100 | 2.70E-76 |
|  |  |  |  | Pfam | MmeI_N | MmeI, N-terminal domain | - | - | 1.80E-26 |
|  |  |  |  | Pfam | MmeI_Mtase | MmeI, DNA-methyltransferase domain | - | - | 4.50E-83 |
|  |  |  |  | Pfam | MmeI_hel | MmeI, helicase spacer domain | - | - | 4.30E-41 |
|  |  |  |  | Pfam | MmeI_TRD | MmeI, target recognition domain | - | - | 5.40E-63 |
|  |  |  |  | Pfam | MmeI_C | MmeI, C-terminal domain | - | - | 1.20E-17 |
| B7017_p0197 | AIW55246 | DNA methylase | N6_N4_Mtase | HHpred | 1G60_A | Adenine-specific Methyltransferase MboIIA | - | 99.63 | 1.70E-14 |
|  |  |  |  | Pfam | N6_N4_Mtase | DNA methylase | - | - | 5.70E-16 |
| B7017_p0202 | AIW55251 | AAA ATPase | DnaA | Phyre2 | c1w5s-B | Origin recognition complex subunit 2 orc2 | 99.9 | - | - |
|  |  |  |  | HHpred | 3PFI_B | Holliday junction ATP-dependent DNA helicase RuvB | - | 99.82 | 1.50E-16 |
|  |  |  |  | Pfam | AAA_16 | AAA ATPase domain | - | - | 3.50E-11 |
|  |  |  |  | Pfam | Bac_DnaA | Bacterial DnaA protein |  |  | 9.20E-11 |
| B7017_p0224 | AIW55273 | pentapeptide repeat protein | PRP/ubiquitin-protein ligase SopA/YjbI | Phyre2 | c4yc5-A | Beta1 | 99.8 | - | - |
|  |  |  |  | HHpred | 5JW7_A | E3 ubiquitin-protein ligase SopA | - | 99.67 | 9.50E-17 |
|  |  |  |  | Pfam | Penta-peptide | Pentapeptide repeats (8 copies) | - | - | 1.90E-13 |

Table S3. pMP7017-expressed Type II TA systems: activity and process inhibition

| Toxinsuper-family | Toxin | Activity | Inhibition | Gene pair(s) |
| --- | --- | --- | --- | --- |
| AtaT/TacT | TacT | Acetylation of elongation tRNAs | Translation elongation | B7017_p0101-B7017_p0102 |
| HipA | HipA | Phosphorylation of Glutamyl-tRNA-synthetase | Translation elongation | B7017_p0120-B7017_p0121 |
| ParE/RelE | RelE | Ribosome-dependent mRNA cleavage | Translation elongation | B7017_p0014-B7017_p0015 |
|  |  |  |  | B7017_p0164-B7017_p0165 |

Table S4. Analysis of CRISPR spacer targets of the pMP7017 Type IE CRIPSR-cas system

| Spacer ID | Spacer sequence 5' - 3' | Target | Target | Accession | Coverage (%) |
| --- | --- | --- | --- | --- | --- |
| Spacer1 | tttccgccgtaatccgattccgcgtgcggaatc | *Microbacterium* phage Morrill | terminase | UQT01735.1 | 45.5 |
| Spacer2 | gaaaattaacccaattaaaccaattgttcctca | *Mycobacterium* phage Superphikiman | Intergenic region | - | 48.5 |
| Spacer4 | ctattcagaaaatgcgatgtggtcggtagggaa | Mycobacterium phage Anaya | NrdH-like glutaredoxin | AEK08028.1 | 48.5 |
| Spacer5 | cgaatcccagggccatcacggccgtggtccggg | *Mycobacterium* phage KayaCho | hypothetical protein | AGT12995.1 | 54.5 |
| Spacer6 | cgtgggagcagaccaaccccgcctatagggtg | *Streptomyces* phage Ibantik | DNA polymerase I | AWN05242.1 | 45.5 |
| Spacer7 | tggaatcgaactcatcatttccaatccctgctt | *Rhodococcus* phage Grayson | hypothetical protein | AWN04483.1 | 48.5 |
| Spacer8 | ctgggaacacacgcaagacaaacgcgacctgag | *Arthrobacter* phage Persistence | HNH endonuclease | QWY79717 | 48.5 |
| Spacer9 | tttgggtggaatcgtctccgccggtggtgtttt | Propionibacterium phage SKKY | minor tail protein | AKO60425 | 51.5 |
| Spacer10 | taggcgacgggctcgacctgaaacagatcgaat | *Siphoviridae* sp. isolate ct0MU3 | Integrase | DAJ53678.1 | 100.0 |
| Spacer13 | ccagaggggcaacgtggcggtcgacaactacac | *Microbacterium* phage Alakazam | major tail protein | QIN93839 | 54.5 |
| Spacer15 | agtcggcatgcttgataatctcatcacgctggta | Bixzunavirus I3 | hypothetical protein | QWT30384.1 | 45.5 |
| Spacer16 | gctgacgccgaggagaaacagatcaaggaactg | *Mycobacterium* phage Raymond7 | type 1 restriction enzyme | QKY79207 | 57.6 |
| Spacer17 | gaacggggaatggtacaccacactgccattgca | *Streptomyces* phage Yaboi | LysM-like | AYB70884.1 | 48.5 |
| Spacer18 | gattctgccgatcgacattccgtcgagcaggac | *Siphoviridae* sp. isolate ctleM7 | hypothetical protein | DAP75288.1 | 100.0 |
| Spacer19 | cattcccgtgcccaatatgatcataggggaaaa | *Arthrobacter* phage BenitoAntonio | membrane protein | QXO13000.1 | 48.5 |
| Spacer21 | tcttggcgacgatggcacacgccaccagtgcag | *Mycobacterium* phage Scorpia | head-to-tail adaptor | "QBP29015.1 | 48.5 |
| Spacer22 | cgtgccgtcacggatatgacccgcatcagcgga | *Mycobacterium* phage Sandalphon | tape measure protein | AXC34923.1 | 45.5 |
| Spacer24 | ggggacgggaaagacagtcctgcgaatcatatg | *Streptomyces* phage Mildred21 | hypothetical protein | ASR75603.1 | 51.5 |
| Spacer25 | ccggcgttcatacaccatcggcatatcgacatg | *Gordonia* phage Trax | Cro protein | QDM55928.1 | 48.5 |

Table S5. pMP7017-expressed RNAs

| pMP7017 tRNAs | | | | | Identical hits RNAcentral | | | Host tRNAs | | |
| --- | --- | --- | --- | --- | --- | --- | --- | --- | --- | --- |
| Family | RNA | Annotation | From | To | Accession | *Bifidobacterium* species | Product | Locus_tag | Product | %ID |
| tRNA | tRNA1 | tRNA-Tyr | 121,375 | 121,456 | URS0000851EBE_1679 | *longum* subsp. *longum* | tRNA-Tyr | Bbr_TRNA10 | tRNA-Tyr | 81.93 |
|  | tRNA2 | tRNA-OTHER | 121,645 | 121,714 | URS00019FB93B_1679 | *longum* subsp. *longum* | tRNA-OTHER | - | - | - |
|  | tRNA3 | tRNA-Pro | 121,962 | 122,035 | URS0001E78DE5_1679 | *longum* subsp. *longum* | tRNA-Pro | Bbr_TRNA33 | tRNA-Pro | 85.19 |
|  | tRNA4 | tRNA-Gly | 122,117 | 122,190 | URS0001FADD45_1679 | *longum* subsp. *longum* | tRNA-Gly | Bbr_TRNA4 | tRNA-Gly | 73.97 |
|  | tRNA5 | tRNA-Lys | 122,331 | 122,402 | URS000043C33A_1161743 | *longum* subsp. *longum* 44B | tRNA-Lys | - | - | - |
|  | tRNA6 | tRNA-Phe | 122,566 | 122,640 | URS0001E82FD1_1679 | *longum* subsp. *longum* | tRNA-Phe | - | - | - |
|  | tRNA7 | tRNA-Arg | 122,967 | 123,041 | URS00008407BD_1681 | *bifidum* | tRNA-Arg | - | - | - |
|  | tRNA8 | tRNA-OTHER | 123,211 | 123,286 | URS00027B97D0_1681 | *bifidum* | tRNA-Leu | - | - | - |
|  | tRNA9 | tRNA-Arg | 123,429 | 123,503 | URS000064D7AA_1681 | *bifidum* | tRNA-Arg | Bbr_TRNA9 | tRNA-Lys | 90.00 |
|  | tRNA10 | tRNA-Ser | 123,508 | 123,585 | URS00027BE57C_1681 | *bifidum* | tRNA-Ser | - | - | - |
|  | tRNA11 | tRNA-Ser | 123,665 | 123,752 | URS00007C800C_1685 | *breve* | tRNA-Ser | - | - | - |
|  | tRNA12 | tRNA-Gln | 123,871 | 123,941 | URS000085CE88_1365967 | *breve* MCC 1454 | tRNA-Gln | Bbr_TRNA22 | tRNA-Gln | 86.11 |
|  | tRNA13 | tRNA-Cys | 123,949 | 124,022 | URS0001FE5166_1679 | *longum* subsp. *longum* | tRNA-Cys | - | - | - |
|  | tRNA14 | tRNA-Gln | 124,028 | 124,098 | URS00007C83EE_1685 | *breve* | tRNA-Gln | - | - | - |
|  | tRNA15 | tRNA-Arg | 124,133 | 124,207 | URS000085C3A6_1365967 | *breve* MCC 1454 | tRNA-Arg | Bbr_TRNA36 | tRNA-Arg | 78.38 |
|  | tRNA16 | tRNA-Gly | 124,463 | 124,534 | URS000084D80A_1365967 | *breve* MCC 1454 | tRNA-Gly | Bbr_TRNA13 | tRNA-Gly | 84.85 |
|  | tRNA17 | tRNA_leu | 124,947 | 125,023 | URS000086426E_1681 | *bifidum* | tRNA_leu | - | - | - |
|  | tRNA18 | tRNA-OTHER | 125,284 | 125,360 | URS00027A4040_1681 | *bifidum* | tRNA-OTHER | - | - | - |
|  | tRNA19 | tRNA-Thr | 125,493 | 125,565 | URS0000D2026C_1679 | *longum* subsp. *longum* | tRNA-Thr | Bbr_TRNA38 | tRNA-Asp | 76.19 |
|  | tRNA20 | tRNA-Ala | 125,731 | 125,803 | URS0000850594_1679 | *longum* subsp. *longum* | tRNA-Ala | Bbr_TRNA53 | tRNA-Ala | 87.84 |
| Novel sRNA | tmRNA | tmRNA | 120,876 | 121,272 | URS00026BD4EE_1681 | *bifidum* | tmRNA | - | - | - |
|  | manA | manA | 122,711 | 122,830 | URS0000D6BB58_12908 | unclassified sequence | manA RNA | - | - | - |

%ID = Percent Identity.

Table S6. Comparison of codon usage frequencies

| AA | Codon | *B. breve* JCM7017 | pMP7017 | B. longum subsp. longumNCIMB8809 |
| --- | --- | --- | --- | --- |
| Ala | GCG | 0.23 | 0.33 | 0.29 |
| Ala | GCA | 0.13 | 0.16 | 0.21 |
| Ala | GCT | 0.14 | 0.08 | 0.18 |
| Ala | GCC | 0.5 | 0.43 | 0.32 |
| Cys | TGT | 0.18 | 0.18 | 0.32 |
| Cys | TGC | 0.82 | 0.82 | 0.68 |
| Asp | GAT | 0.44 | 0.32 | 0.45 |
| Asp | GAC | 0.56 | 0.68 | 0.55 |
| Glu | GAG | 0.55 | 0.43 | 0.45 |
| Glu | GAA | 0.45 | 0.57 | 0.55 |
| Phe | TTT | 0.17 | 0.09 | 0.23 |
| Phe | TTC | 0.83 | 0.91 | 0.77 |
| Gly | GGG | 0.07 | 0.15 | 0.14 |
| Gly | GGA | 0.11 | 0.22 | 0.21 |
| Gly | GGT | 0.22 | 0.14 | 0.21 |
| Gly | GGC | 0.6 | 0.5 | 0.45 |
| His | CAT | 0.48 | 0.48 | 0.5 |
| His | CAC | 0.52 | 0.52 | 0.5 |
| Ile | ATA | 0.05 | 0.12 | 0.15 |
| Ile | ATT | 0.29 | 0.15 | 0.28 |
| Ile | ATC | 0.66 | 0.73 | 0.57 |
| Lys | AAG | 0.78 | 0.6 | 0.69 |
| Lys | AAA | 0.22 | 0.4 | 0.31 |
| Leu | TTG | 0.16 | 0.18 | 0.16 |
| Leu | TTA | 0.01 | 0.01 | 0.05 |
| Leu | CTG | 0.47 | 0.37 | 0.33 |
| Leu | CTA | 0.03 | 0.04 | 0.08 |
| Leu | CTT | 0.1 | 0.09 | 0.14 |
| Leu | CTC | 0.22 | 0.3 | 0.23 |
| Asn | AAT | 0.31 | 0.19 | 0.37 |
| Asn | AAC | 0.69 | 0.81 | 0.63 |
| Pro | CCG | 0.54 | 0.5 | 0.44 |
| Pro | CCA | 0.09 | 0.15 | 0.25 |
| Pro | CCT | 0.12 | 0.07 | 0.15 |
| Pro | CCC | 0.25 | 0.28 | 0.17 |
| Gln | CAG | 0.79 | 0.68 | 0.45 |
| Gln | CAA | 0.21 | 0.32 | 0.55 |
| Arg | AGG | 0.04 | 0.12 | 0.12 |
| Arg | AGA | 0.03 | 0.07 | 0.08 |
| Arg | CGG | 0.1 | 0.19 | 0.2 |
| Arg | CGA | 0.08 | 0.13 | 0.21 |
| Arg | CGT | 0.28 | 0.17 | 0.15 |
| Arg | CGC | 0.46 | 0.32 | 0.25 |
| Ser | AGT | 0.05 | 0.08 | 0.07 |
| Ser | AGC | 0.2 | 0.34 | 0.18 |
| Ser | TCG | 0.21 | 0.17 | 0.29 |
| Ser | TCA | 0.06 | 0.09 | 0.18 |
| Ser | TCT | 0.08 | 0.05 | 0.11 |
| Ser | TCC | 0.4 | 0.29 | 0.17 |
| Thr | ACG | 0.24 | 0.3 | 0.34 |
| Thr | ACA | 0.07 | 0.12 | 0.18 |
| Thr | ACT | 0.12 | 0.06 | 0.14 |
| Thr | ACC | 0.57 | 0.52 | 0.33 |
| Val | GTG | 0.51 | 0.38 | 0.39 |
| Val | GTA | 0.08 | 0.1 | 0.13 |
| Val | GTT | 0.1 | 0.1 | 0.2 |
| Val | GTC | 0.3 | 0.42 | 0.28 |
| Tyr | TAT | 0.37 | 0.29 | 0.39 |
| Tyr | TAC | 0.63 | 0.71 | 0.61 |

##### Codons with low frequency (<10%) are highlighted in pink. The most preferred codon for each amino acid is highlighted in blue. Methionine, Tyrosine and stop codons are not included.

Table S7. Down-regulated *B. breve* UCC2003 chromosomal genes in the presence of pMP7017

| Protein family | Pathway | Locus-tag/ Symbol | KO | description | COG | fc | Adj. P-value |
| --- | --- | --- | --- | --- | --- | --- | --- |
| Cellular Processes | Polysaccharide biosynthesis | Bbr_0442 | - | Glycosyltransferase polysaccharide | COG0438 | 3.29 | 2.24E-07 |
|  |  | Bbr_0443 | - | Glycosyltransferase polysaccharide | COG0438 | 10.95 | 1.96E-23 |
|  |  | Bbr_0445 | - | Glycosyltransferase polysaccharide | [COG0463](https://www.ncbi.nlm.nih.gov/Structure/cdd/cddsrv.cgi?ascbin=8&maxaln=10&seltype=2&uid=COG0463) | 3.23 | 1.12E-18 |
|  |  | Bbr_0446 | - | Acetyltransferase involved in polysaccharide biosynthesis | COG0110 | 2.67 | 8.67E-11 |
|  |  | Bbr_0447 | - | Polysaccharide pyruvyl transferase | - | 2.96 | 7.28E-20 |
|  |  | Bbr_0448 | - | Glycosyltransferase polysaccharide | [COG0463](https://www.ncbi.nlm.nih.gov/Structure/cdd/cddsrv.cgi?ascbin=8&maxaln=10&seltype=2&uid=COG0463) | 2.88 | 1.76E-16 |
|  |  | Bbr_0450 | - | Membrane spanning protein, polysaccharide biosynthesis | - | 2.20 | 1.06E-05 |
|  |  | pslL | K21005 | polysaccharide biosynthesis protein PslL | COG3594 | 2.69 | 8.42E-07 |
| Environmental Information Processing | Extracellular structures | Bbr_0113 | K21449 | trimeric autotransporter adhesin, SpaA domain | COG4932 | 2.85 | 1.44E-21 |
|  |  | Bbr_0114 | - | Iso_D2_wall_anc | COG4932 | 15.34 | 2.35E-117 |
|  |  | Bbr_1888 | - | Uncharacterized surface anchored protein | COG4932 | 2.01 | 7.86E-09 |
|  | Membrane transport | malE | K15770 | maltooligosaccharide transport system | COG2182 | 2.99 | 3.63E-21 |
|  |  | malC | K15772 | maltooligosaccharide transport system permease | COG1175 | 2.82 | 8.21E-23 |
|  |  | malG | K15771 | maltooligosaccharide transport system permease | COG3833 | 2.95 | 1.97E-39 |
|  |  | Bbr_0273 | K01990 | ABC-2 type transporter | [COG1131](https://www.ncbi.nlm.nih.gov/Structure/cdd/cddsrv.cgi?ascbin=8&maxaln=10&seltype=2&uid=COG1131) | 7.67 | 7.99E-34 |
|  |  | Bbr_0274 | K01990 | ABC-2 type transporter | COG1682 | 3.77 | 3.40E-30 |
|  |  | Bbr_1501 | K01990 | ABC-2 type transporter | COG1131 | 2.44 | 1.29E-17 |
|  |  | Bbr_1836 | K10439 | ribose transport system substrate-binding protein | COG1879 | 2.61 | 5.63E-13 |
| Metabolism | Carbohydrate | agl4 | K01187 | alpha glucosidase | COG0366 | 2.22 | 1.29E-09 |
|  |  | apuB | K01200 | Amylopullulanase | COG1523 | 2.66 | 1.44E-21 |
|  | Fatty acid | accD | K01966 | propionyl-CoA carboxylase | COG4799 | 2.10 | 1.45E-08 |
|  | Amino acid transport and metabolism | NA | - | tRNA Ile | - | 3.04 | 1.45E-06 |
|  |  | NA | - | tRNA-Ala | - | 2.96 | 3.48E-06 |
|  |  | NA | - | tRNA-Ala | - | 2.10 | 1.57E-04 |
|  |  | NA | - | tRNA-Val | - | 2.77 | 4.00E-02 |
|  |  | NA | - | tRNA-Lys | - | 2.03 | 1.37E-05 |
| Mobilome | prophages, transposons | Bbr_0465 | - | Bacteriophage abortive infection | - | 2.16 | 3.16E-02 |
| Unknown | Unknown | Bbr_0121 | - | TraX protein acetylation of pro-pilin | - | 2.16 | 1.69E-17 |
|  |  | Bbr_0449 | - | hypothetical protein | - | 2.92 | 2.30E-07 |
|  |  | Bbr_1748 | - | Acetyltransferase | COG0456 | 4.08 | 1.01E-20 |

KO = KEGG Orthology, COG = Clusters of Orthologous Genes, fc = fold change, Adj. P-value = Adjusted P-value.

Table S8. Gene Ontology enrichment analyses of down-regulated *B. breve* UCC2003 chromosomal genes in the presence of pMP7017

| Pathway | nGenes | Pathway Genes | Fold Enrichment | Enrichment FDR | Gene/ symbol | | | | | |
| --- | --- | --- | --- | --- | --- | --- | --- | --- | --- | --- |
| Glycosyl transferases | 9 | 10 | 187.5 | 1.88E-19 | BBR_0451 | Bbr_0442 | Bbr_0443 | Bbr_0445 | Bbr_0446 | Bbr_0447 |
|  |  |  |  |  | Bbr_0448 | Bbr_0449 | Bbr_0450 |  |  |  |
| Carbohydrate metabolism | 11 | 117 | 19.6 | 2.13E-11 | agl4 | apuB | Bbr_0442 | Bbr_0443 | Bbr_0445 | Bbr_0446 |
|  |  |  |  |  | Bbr_0447 | Bbr_0448 | Bbr_0449 | Bbr_0450 | BBR_0451 |  |
| Polysaccharide biosynthesis protein | 4 | 5 | 166.7 | 2.24E-08 | Bbr_0442 | Bbr_0443 | Bbr_0445 | Bbr_0446 |  |  |
|  |  |  |  |  |  |  |  |  |  |  |
| Cna protein B-type domain | 3 | 5 | 125.0 | 9.30E-06 | Bbr_0113 | Bbr_0114 | Bbr_1888 |  |  |  |
|  |  |  |  |  |  |  |  |  |  |  |
| Transmembrane | 11 | 461 | 5.0 | 2.30E-05 | Bbr_0113 | Bbr_0114 | malC | malG | Bbr_0121 | apuB |
|  |  |  |  |  | Bbr_0274 | Bbr_0449 | Bbr_0450 | BBR_0451 | Bbr_1888 |  |
| Transferase | 7 | 279 | 5.2 | 1.08E-03 | apuB | Bbr_0443 | Bbr_0445 | Bbr_0446 | Bbr_0448 | BBR_0451 |
|  |  |  |  |  | Bbr_1748 |  |  |  |  |  |
| Alpha amylase, catalytic domain | 2 | 13 | 32.1 | 4.28E-03 | agl4 | apuB |  |  |  |  |
|  |  |  |  |  |  |  |  |  |  |  |
| Glycosidase | 2 | 45 | 9.3 | 3.62E-02 | agl4 | apuB |  |  |  |  |
|  |  |  |  |  |  |  |  |  |  |  |

Table S9. Up-regulated *B. breve* UCC2003 chromosomal genes in the presence of pMP7017

| Protein family | Pathway | Locus-tag/ Symbol | KO | Pathway/description | COG | fc | Adj. P- value |
| --- | --- | --- | --- | --- | --- | --- | --- |
| Environmental Information Processing | Signal transduction | Bbr_0062 | - | Membrane associated serine protease, rhomboid family | [COG0705](https://www.ncbi.nlm.nih.gov/Structure/cdd/cddsrv.cgi?ascbin=8&maxaln=10&seltype=2&uid=COG0705) | 2.82 | 1.60E-05 |
|  |  | Bbr_1060 | - | Signal transduction histidine kinase | COG4585 | 2.65 | 1.66E-04 |
| Cellular Processes | Biofilm formation | pelF | K21011 | biofilm biosynthesis glycosyltransferase | COG0438 | 3.37 | 7.88E-05 |
|  |  | pelG | K21012 | polysaccharide exporter | COG4267 | 3.13 | 6.41E-05 |
|  |  | Bbr_0046 | K06330 | Spore coat protein CotH | [COG5337](https://www.ncbi.nlm.nih.gov/Structure/cdd/cddsrv.cgi?ascbin=8&maxaln=10&seltype=2&uid=COG5337) | 2.82 | 4.67E-09 |
|  |  | Bbr_0047 | - | CYTH-like phosphatase | - | 2.78 | 7.60E-05 |
|  |  | Bbr_0048 | - | Mg2+ transport | - | 2.07 | 1.82E-11 |
| Environmental Information Processing | Membrane transport | Permease | K02004 | ABC-type transport system, involved in lipoprotein release, permease component | COG4591 | 2.13 | 2.67E-03 |
|  |  | cldF | K10241 | cellobiose transport system permease protein | COG1175 | 2.41 | 7.28E-20 |
|  |  | cldG | K10242 | cellobiose transport system permease protein | COG0395 | 2.87 | 2.98E-16 |
|  |  | Bbr_1341 | K06895 | L-lysine exporter family protein LysE/ArgO | COG1279 | 3.37 | 2.04E-11 |
|  |  | Bbr_1858 | K10117 | transport system permease protein | COG0395 | 2.22 | 8.18E-08 |
|  |  | Bbr_1862 | K10117 | transport system substrate-binding protein | COG1653 | 2.13 | 5.77E-17 |
|  |  | Bbr_0259 | K01990 | ABC-2 type transporter | COG1134 | 2.09 | 9.04E-03 |
|  | Signal transduction | Bbr_0859 | K08217 | Macrolide-efflux protein | COG0477 | 2.13 | 6.26E-09 |
|  |  | Bbr_1049 | - | VanZ family protein | [COG4767](https://www.ncbi.nlm.nih.gov/Structure/cdd/cddsrv.cgi?ascbin=8&maxaln=10&seltype=2&uid=COG4767) | 2.49 | 7.39E-08 |
|  |  | Bbr_1065 | - | ABC-type multidrug transport system, permease | COG0577 | 2.74 | 1.83E-13 |
|  |  | Bbr_1071 | K01992 | ABC-type multidrug transport system, permease | COG0842 | 3.05 | 8.75E-05 |
|  |  | Bbr_1483 | - | Multidrug resistance protein B | COG0842 | 3.52 | 1.62E-04 |
| General function prediction only | General function prediction only | Bbr_1270 | - | LysM domain protein | [COG3170](https://www.ncbi.nlm.nih.gov/Structure/cdd/cddsrv.cgi?ascbin=8&maxaln=10&seltype=2&uid=COG3170) | 4.73 | 1.23E-31 |
|  |  | Bbr_1290 | - | Predicted metal-dependent hydrolase, TIM-barrel fold | COG3618 | 4.14 | 1.70E-17 |
|  |  | Bbr_1515 | - | ATP-binding protein of ABC transporter system | [COG0488](https://www.ncbi.nlm.nih.gov/Structure/cdd/cddsrv.cgi?ascbin=8&maxaln=10&seltype=2&uid=COG0488) | 2.08 | 3.46E-07 |
|  |  | Bbr_1741 | - | Predicted metal-dependent hydrolase, TIM-barrel fold | COG3618 | 2.32 | 2.12E-03 |
| Genetic Information Processing | Transcription factors | Bbr_0032 | K05499 | LacI family transcriptional regulator | - | 2.18 | 5.06E-08 |
|  |  | Bbr_0208 | - | Transcriptional regulator, TetR family | - | 2.27 | 4.94E-15 |
|  |  | Bbr_0849 | K15545 | NagC/XylR-type transciptional regulator | COG1940 | 2.76 | 9.62E-14 |
|  |  | Bbr_1309 | - | DNA-binding transcriptional regulator, MarR family | COG1846 | 2.22 | 1.29E-09 |
|  |  | Bbr_1340 | K18907 | GntR family transcriptional regulator, regulator for abcA and norABC | COG1167 | 2.86 | 1.24E-13 |
|  |  | cspA | K03704 | Cold shock protein | COG1278 | 2.53 | 9.04E-14 |
|  |  | Bbr_1389 | - | Transcription antiterminator, BglG family | [COG3711](https://www.ncbi.nlm.nih.gov/Structure/cdd/cddsrv.cgi?ascbin=8&maxaln=10&seltype=2&uid=COG3711) | 2.34 | 1.71E-02 |
|  |  | Bbr_1509 | - | Transcription regulator, HTH_36 domain | - | 3.96 | 1.02E-10 |
|  |  | Bbr_1510 | - | WhiB family transcriptional regulator | - | 2.98 | 1.53E-02 |
|  |  | Bbr_1519 | - | DNA-binding transcriptional regulator, MerR family | [COG0789](https://www.ncbi.nlm.nih.gov/Structure/cdd/cddsrv.cgi?ascbin=8&maxaln=10&seltype=2&uid=COG0789) | 2.21 | 8.34E-05 |
|  |  | Bbr_1523 | - | DNA-binding transcriptional regulator, MerR family | [COG0789](https://www.ncbi.nlm.nih.gov/Structure/cdd/cddsrv.cgi?ascbin=8&maxaln=10&seltype=2&uid=COG0789) | 3.73 | 1.28E-19 |
|  | Transcription machinery | Bbr_1068 | - | RNA polymerase sigma-E factor | COG1595 | 4.18 | 3.79E-03 |
|  | Translation factors | Bbr_1079 | - | Ribosome-associated factor Y | [COG2197](https://www.ncbi.nlm.nih.gov/entrez/query.fcgi?cmd=Search&doptcmdl=GenPept&db=cdd&term=COG2197) | 2.19 | 1.48E-05 |
|  |  | infC | K02520 | Translation initiation factor IF-3 | [COG0290](https://www.ncbi.nlm.nih.gov/Structure/cdd/cddsrv.cgi?ascbin=8&maxaln=10&seltype=2&uid=COG0290) | 2.29 | 9.80E-24 |
|  |  | Bbr_1516 | - | Acetyltransferase (GNAT) family | COG0456 | 2.24 | 1.96E-08 |
|  | Homologous recombination | recG | K03655 | ATP-dependent DNA helicase RecG | COG1200 | 3.31 | 5.23E-04 |
|  |  | Bbr_1113 | - | site-specific tyrosine recombinase XerC | [COG4973](https://www.ncbi.nlm.nih.gov/research/cog/cog/COG4973/) | 25.58 | 5.27E-19 |
|  |  | topB | K03169 | DNA topoisomerase III | COG0550 | 4.40 | 2.98E-09 |
|  |  | Bbr_1548 | - | site-specific tyrosine recombinase XerC | [COG0582](https://www.ncbi.nlm.nih.gov/Structure/cdd/cddsrv.cgi?ascbin=8&maxaln=10&seltype=2&uid=COG0582) | 14.11 | 9.14E-21 |
|  | Folding, sorting and degradation | Bbr_1222 | - | MutT/nudix family phosphohydrolase | COG1051 | 3.30 | 3.34E-11 |
|  | Replication and repair | Bbr_0585 | K03502 | DNA polymerase V | COG0389 | 3.78 | 8.15E-21 |
|  |  | lexA | K01356 | SOS-response transcriptional repressor LexA (RecA-mediated autopeptidase) | [COG1974](https://www.ncbi.nlm.nih.gov/Structure/cdd/cddsrv.cgi?ascbin=8&maxaln=10&seltype=2&uid=COG1974) | 2.25 | 3.43E-11 |
|  |  | parB1 | K03497 | ParB family transcriptional regulator | COG1475 | 3.31 | 6.15E-04 |
|  | Intracellular trafficking, secretion | Bbr_1128 | - | Predicted membrane metal-binding protein | [COG0658](https://www.ncbi.nlm.nih.gov/Structure/cdd/cddsrv.cgi?ascbin=8&maxaln=10&seltype=2&uid=COG0658) | 14.38 | 3.65E-21 |
|  |  | Relaxase | - | relaxase | COG3843 | 3.82 | 1.52E-02 |
|  |  | Bbr_1545 | - | MobC | COG3843 | 3.01 | 4.23E-02 |
| Metabolism | terpenoids and polyketides | Bbr_0205 | - | type I polyketide synthase | - | 2.18 | 9.55E-09 |
|  | cofactors and vitamins | Bbr_0209 | - | Phosphopantetheinyl transferase | - | 2.30 | 3.01E-10 |
|  |  | Bbr_1717 | - | Pimeloyl-ACP methyl ester carboxylesterase | [COG0596](https://www.ncbi.nlm.nih.gov/Structure/cdd/cddsrv.cgi?ascbin=8&maxaln=10&seltype=2&uid=COG0596) | 3.28 | 6.74E-04 |
|  |  | Bbr_1718 | - | NAD(P)H-dependent FMN reductase | [COG0431](https://www.ncbi.nlm.nih.gov/Structure/cdd/cddsrv.cgi?ascbin=8&maxaln=10&seltype=2&uid=COG0431) | 4.70 | 1.70E-04 |
|  | Amino acid transport and metabolism | Bbr_0342 | K02055 | Spermidine/putrescine-binding periplasmic protein | COG0687 | 2.70 | 4.00E-04 |
|  |  | BBR_0600 | - | Aspartate/methionine/tyrosine aminotransferase | - | 4.11 | 3.29E-03 |
|  |  | Bbr_1061 | - | Nucleotide pyrophosphatase | COG2197 | 2.06 | 3.51E-02 |
|  | other amino acids | oppD3 | K13892 | glutathione transport system ATP-binding protein | COG1123 | 2.75 | 3.56E-09 |
|  |  | oppC2 | K13891 | glutathione transport system permease protein | COG1173 | 2.43 | 6.01E-04 |
|  |  | bgl4 | K05349 | beta-glucosidase | COG1472 | 2.30 | 4.25E-04 |
|  | Transfer RNA Biogenesis | truA | K06173 | tRNA pseudouridine38-40 synthase | COG0101 | 2.83 | 1.38E-12 |
|  |  | dtd | K07560 | D-aminoacyl-tRNA deacylase | COG1490 | 2.92 | 6.76E-14 |
|  | Carbohydrate | lacZ3 | - | Beta-galactosidase/beta-glucuronidase | COG2723 | 2.32 | 3.44E-04 |
|  |  | BBR_1143 | - | Alpha-glucosidase | COG1501 | 2.13 | 1.94E-05 |
|  |  | cwlO | K21471 | peptidoglycan DL-endopeptidase CwlO | [COG0791](https://www.ncbi.nlm.nih.gov/Structure/cdd/cddsrv.cgi?uid=COG0791) | 3.68 | 1.27E-03 |
|  |  | acyP | K01512 | Acylphosphatase | [COG1254](https://www.ncbi.nlm.nih.gov/Structure/cdd/cddsrv.cgi?ascbin=8&maxaln=10&seltype=2&uid=COG1254) | 2.45 | 6.06E-03 |
|  |  | dapA3 | K22397 | Dihydrodipicolinate synthase | COG0329 | 3.45 | 2.71E-19 |
|  |  | dapA4 | K22397 | Dihydrodipicolinate synthase | COG0329 | 2.18 | 6.96E-05 |
|  |  | Bbr_1291 | K18333 | L-fucose dehydrogenase | COG1028 | 4.38 | 5.82E-28 |
| Mobilome | prophages, transposons | BBR_0024 | - | Transposase | - | 2.15 | 1.16E-05 |
|  |  | BBR_0159 | - | Transposase | - | 3.66 | 1.30E-03 |
|  |  | tnpY | - | Transposase | - | 2.41 | 2.65E-10 |
|  |  | Bbr_0302 | - | Transposase | - | 2.71 | 7.26E-03 |
|  |  | Bbr_0457 | - | IS21 family transposase | - | 2.87 | 1.60E-05 |
|  |  | Bbr_1401 | - | transposase | - | 3.73 | 3.59E-03 |
|  |  | Bbr_1404 | - | Transposase | [COG4584](https://www.ncbi.nlm.nih.gov/Structure/cdd/cddsrv.cgi?ascbin=8&maxaln=10&seltype=2&uid=COG4584) | 2.20 | 3.67E-02 |
| Secretion system | Type II secretion system | tadA | K02283 | Pilus assembly ATPase | COG4962 | 9.35 | 2.90E-03 |
|  |  | tadB | K12510 | tight adherence pilus assembly protein | COG4965 | 4.00 | 4.47E-02 |
|  |  | tadF | - | Pilin precursor | - | 5.21 | 2.57E-02 |
| Unknown | Unknown | Bbr_0055 | - | Conserved hypothetical protein | - | 2.69 | 1.45E-08 |
|  |  | Bbr_0145 | - | Conserved hypothetical protein | - | 2.03 | 1.49E-02 |
|  |  | Bbr_0202 | - | Conserved hypothetical protein | - | 3.02 | 1.23E-05 |
|  |  | Bbr_0218 | - | hypothetical protein | - | 2.17 | 1.94E-03 |
|  |  | Bbr_0264 | - | hypothetical protein | - | 2.47 | 2.72E-24 |
|  |  | Holin | - | hypothetical phage protein | - | 3.84 | 4.35E-03 |
|  |  | Bbr_0300 | - | hypothetical secreted protein | - | 2.47 | 1.60E-18 |
|  |  | Bbr_0315 | - | hypothetical phage protein (flanking tRNA10) | - | 3.32 | 5.33E-04 |
|  |  | Bbr_0316 | - | hypothetical phage protein (flanking tRNA10) | - | 3.41 | 4.52E-02 |
|  |  | Bbr_0317 | - | hypothetical phage protein | - | 4.98 | 2.58E-08 |
|  |  | Bbr_0318 | - | hypothetical protein | - | 3.60 | 9.24E-07 |
|  |  | Bbr_0319 | - | phage transcription regulator protein | - | 2.27 | 7.83E-11 |
|  |  | Bbr_0320 | - | hypothetical protein | - | 2.44 | 9.87E-12 |
|  |  | Bbr_0337 | - | hypothetical protein | - | 3.86 | 4.96E-02 |
|  |  | Bbr_0362 | - | conserved hypothetical protein (flanking tRNA13) | - | 2.36 | 5.46E-05 |
|  |  | Bbr_0364 | - | hypothetical protein | - | 2.15 | 3.83E-02 |
|  |  | Bbr_0366 | - | Conserved hypothetical secreted protein | - | 2.09 | 9.42E-06 |
|  |  | Bbr_0376 | - | Conserved membrane spanning rhomboid family protein | - | 3.38 | 2.49E-08 |
|  |  | Bbr_0384 | - | Conserved membrane spanning rhomboid family protein | - | 4.37 | 3.41E-37 |
|  |  | Bbr_0452 | - | hypothetical protein | - | 2.72 | 2.67E-03 |
|  |  | Bbr_0454 | - | conserved hypothetical protein | - | 2.04 | 4.19E-02 |
|  |  | Bbr_0501 | - | conserved hypothetical protein | [COG1721](https://www.ncbi.nlm.nih.gov/Structure/cdd/cddsrv.cgi?ascbin=8&maxaln=10&seltype=2&uid=COG1721) | 3.73 | 1.56E-05 |
|  |  | Bbr_0502 | - | MoxR-like ATPase | COG0714 | 2.61 | 8.84E-11 |
|  |  | Bbr_0572 | - | hypothetical protein | - | 2.59 | 3.11E-02 |
|  |  | Bbr_0591 | - | hypothetical protein | - | 2.42 | 5.35E-03 |
|  |  | Bbr_0592 | - | hypothetical protein | - | 16.44 | 4.91E-02 |
|  |  | Bbr_0593 | - | Conserved hypothetical secreted protein | - | 2.07 | 2.65E-03 |
|  |  | Bbr_0638 | - | conserved hypothetical protein | - | 2.41 | 2.17E-03 |
|  |  | Bbr_0729 | - | Conserved membrane spanning rhomboid family protein | - | 2.51 | 3.15E-03 |
|  |  | Bbr_0836 | - | conserved hypothetical protein | - | 2.37 | 1.79E-08 |
|  |  | Bbr_1058 | - | conserved hypothetical protein | - | 3.92 | 8.18E-08 |
|  |  | Bbr_1069 | - | Conserved membrane spanning rhomboid family protein | - | 4.68 | 7.45E-05 |
|  |  | Bbr_1070 | - | Conserved membrane spanning rhomboid family protein | - | 2.76 | 1.35E-02 |
|  |  | Bbr_1151 | - | hypothetical protein | - | 3.26 | 1.07E-09 |
|  |  | Bbr_1306 | - | Conserved membrane spanning rhomboid family protein | - | 2.21 | 2.11E-09 |
|  |  | Bbr_1308 | - | Conserved membrane spanning rhomboid family protein | - | 2.41 | 7.63E-06 |
|  |  | Bbr_1509a | - | hypothetical protein | - | 4.29 | 3.61E-03 |
|  |  | Bbr_1524 | - | PIN_3 RNAse | - | 3.13 | 2.66E-24 |
|  |  | Bbr_1528 | - | Conserved membrane spanning rhomboid family protein | - | 20.90 | 1.35E-03 |
|  |  | Bbr_1537 | - | hypothetical protein | - | 3.78 | 4.58E-02 |
|  |  | Bbr_1538 | - | hypothetical protein | - | 5.05 | 4.16E-02 |
|  |  | Bbr_1546a | - | hypothetical protein | - | 2.18 | 4.73E-02 |
|  |  | Bbr_1547 | - | hypothetical protein | - | 3.98 | 2.31E-03 |
|  |  | Bbr_1609 | - | hypothetical protein | - | 2.45 | 6.43E-10 |
|  |  | Bbr_1728 | - | hypothetical protein | - | 2.04 | 1.30E-03 |
|  |  | Bbr_1765 | - | Conserved membrane spanning rhomboid family protein | - | 4.25 | 6.85E-15 |
|  |  | Bbr_1772 | - | conserved hypothetical protein | - | 2.86 | 3.72E-15 |
|  |  | Bbr_1861 | - | conserved hypothetical protein | - | 2.32 | 1.14E-18 |

Table S10. Gene Ontology enrichment analyses of up-regulated *B. breve* UCC2003 chromosomal genes in the presence of pMP7017

| Pathway | nGenes | nGenes total gene pool | Fold Enrichment | Enrichment FDR | Gene/ symbol | | | | | | |
| --- | --- | --- | --- | --- | --- | --- | --- | --- | --- | --- | --- |
| Biofilm biosynthesis | 4 | 6 | 44.4 | 2.83E-05 | Bbr_0044 | Bbr_0045 | Bbr_0046 | Bbr_0047 |  |  |  |
| MetI-like superfamily | 2 | 4 | 33.3 | 1.30E-02 | cldF | cldG |  |  |  |  |  |
| Diaminopimelate pathway | 2 | 4 | 33.3 | 1.30E-02 | dapA3 | dapA4 |  |  |  |  |  |
| Tad-like protein | 3 | 8 | 25.0 | 2.37E-03 | tadA | tadB | tadF |  |  |  |  |
| Integrase core domain | 4 | 20 | 13.3 | 2.46E-03 | Bbr_0159 | Bbr_0302 | Bbr_0457 | Bbr_1404 |  |  |  |
| Fatty acid metabolism, and phospholipase/carboxylesterase | 7 | 51 | 9.2 | 1.98E-04 | Bbr_0205 | Bbr_0209 | dapA3 | Bbr_1290 | Bbr_1291 | dapA4 | Bbr_1741 |
|  |  |  |  |  |  |  |  |  |  |  |  |
| SOS response | 1 | 8 | 8.3 | 1.85E-01 | lexA |  |  |  |  |  |  |
| Transmembrane, and ABC transporter | 13 | 114 | 7.6 | 7.22E-07 | Bbr_0044 | Bbr_0045 | Bbr_0046 | Bbr_0047 | Bbr_0062 | Bbr_0208 | tnpY |
|  |  |  |  |  | Bbr_0259 | Bbr_1060 | Bbr_1061 | Bbr_1065 | Bbr_1068 | Bbr_1071 |  |
| Quorum sensing | 2 | 20 | 6.7 | 1.14E-01 | oppD3 | oppC2 |  |  |  |  |  |
| Transcription regulation | 7 | 75 | 6.2 | 1.87E-03 | Bbr_0032 | Bbr_0208 | Bbr_1061 | Bbr_1068 | Bbr_1079 | lexA | Bbr_1340 |
| DNA-binding | 10 | 115 | 5.8 | 1.82E-04 | Bbr_0032 | Bbr_0146 | Bbr_0208 | Bbr_1061 | Bbr_1068 | Bbr_1079 | Bbr_1113 |
|  |  |  |  |  | lexA | Bbr_1340 | Bbr_1548 |  |  |  |  |
| Transport, and signalling | 10 | 183 | 3.6 | 4.38E-03 | cldF | cldG | Bbr_0205 | lacZ3 | Bbr_0342 | Bbr_0849 | oppD3 |
|  |  |  |  |  | oppC2 | Bbr_1858 | Bbr_1862 |  |  |  |  |
| Signal | 5 | 93 | 3.6 | 5.69E-02 | Bbr_0046 | Bbr_0342 | Bbr_1530 | Bbr_1858 | Bbr_1862 |  |  |
| Transport | 5 | 104 | 3.2 | 7.15E-02 | cldF | cldG | oppD3 | oppC2 | Bbr_1858 |  |  |
| Transmembrane | 18 | 461 | 2.6 | 1.87E-03 | Bbr_0045 | Bbr_0062 | cldF | cldG | tadB | tadF | Bbr_0859 |
|  |  |  |  |  | Bbr_1049 | Bbr_1060 | Bbr_1065 | Bbr_1071 | Bbr_1081 | Bbr_1128 | Bbr_1341 |
|  |  |  |  |  | Bbr_1483 | Bbr_1530 | oppC2 | Bbr_1858 |  |  |  |
| Schiff base-forming aldolases | 2 | 7 | 19.0 | 2.62E-02 | dapA3 | dapA4 |  |  |  |  |  |
| Transcription | 7 | 80 | 5.8 | 2.37E-03 | Bbr_0032 | Bbr_0208 | Bbr_1061 | Bbr_1068 | Bbr_1079 | lexA | Bbr_1340 |

Table S11. Prevalence of pMP7017 among metagenomic samples

| Study | Country | NGS platform(s) | Total Samples | Mapped Samples |
| --- | --- | --- | --- | --- |
| Nishijama 2016(PMID: 26951067) | Japan | 454, Ion PGM and illumina Miseq | 400 | 36 |
| DIABIMMUNE cohort(PMID: 30559407) | Russia | Illumina | 89 | 34 |
| DIABIMMUNE cohort(PMID: 30559407) | Finland | Illumina | 102 | 40 |
| DIABIMMUNE cohort(PMID: 30550407) | Estonia | Illumina | 97 | 29 |
| Backhed 2015 (PMID: 25974306) | Sweden | Illumina | 312 | 16 |
| Shao 2019 (PMID: 31534227) | UK | Illumina | 588 | 41 |
| Ferretti 2018, Asnicar 2017 (PMID: 30001516,  PMID: 28144631) | Italy | Illumina | 102 | 6 |
| Wampach 2018 (PMID: 30504906) | Luxembourg | Illumina | 49 | 3 |
| Chu 2017 (PMID: 28112736) | USA | Illumina | 27 | 1 |
|  |  |  |  |  |
|  |  | Total samples | 1846 |  |
|  |  | Mapped samples | 206 |  |
|  |  | >70% mapped genes | 10 |  |
